# HIV-1 Transcription Inhibitor 1E7-03 Decreases Nucleophosmin Phosphorylation

**DOI:** 10.1016/j.mcpro.2022.100488

**Published:** 2022-12-21

**Authors:** Xionghao Lin, Asrar Ahmad, Andrey I. Ivanov, Jyothirmai Simhadri, Songping Wang, Namita Kumari, Tatiana Ammosova, Sergei Nekhai

**Affiliations:** 1 Center for Sickle Cell Disease, College of Medicine, Howard University, Washington, District of Columbia, USA; 2 College of Dentistry, Howard University, Washington, District of Columbia, USA; 3 Department of Microbiology, College of Medicine, Howard University, Washington, District of Columbia, USA; 4 Department of Medicine, College of Medicine, Howard University, Washington, District of Columbia, USA

**Keywords:** ACTN4, alpha-actinin-1, ASL, argininosuccinate lyase, ASPM, abnormal spindle-like microcephaly-associated protein, cART, combination antiretroviral therapy, CDK2, cell cycle-dependent kinase 2, CK2, casein kinase 2, DMSO, dimethyl sulfoxide, EGLN1, Egl-9 Family Hypoxia Inducible Factor 1, Erk/p38, extracellular signal-regulated kinase p38, FA, formic acid, GADD34, growth arrest and DNA damage-inducible protein, HIF-1α, Hypoxia-inducible factor 1α, HIV-1 Vif protein, viral infectivity factor, an HIV-1 accessory protein, HIV-1, human immunodeficiency virus-1, Hsp90, heat shock protein 90, IPA, Ingenuity Pathway Analysis, LC-FT/MS, tandem liquid chromatography-Fourier transform mass spectrometry, MAPK, Mitogen-activated protein kinase, MAP3K4, Mitogen-activated protein kinase kinase kinase 4, MITA, mediator of interferon response factor 3 activation, NFAT, Nuclear factor of activated T cells, NF-κB, nuclear factor kappa-light-chain-enhancer of activated B cell, NPM1, nucleophosmin, OA, okadaic acid, PI3K/Akt, Phosphoinositide 3-kinase/ Ak strain transforming or protein kinase B, PP, protein phosphatase, PPARα/RXRα, peroxisome proliferator-activated receptor α/ retinoid X receptor α, PTM, posttranslational modification, RNR2, ribonucleotide reductase 2, RT, reverse transcription, SAMHD1, SAM domain and HD domain-containing protein 1, SMAD7, Mothers against decapentaplegic homolog 7, STAT5, Signal transducer and activator of transcription 5 TAF4, TAF4, transcription factor TFIID subunit TATA-box-binding protein (TBP)-associated factor 4, TGF-β2, transforming growth factor-beta, TP53, tumor protein p53

## Abstract

Transcription activation of latent human immunodeficiency virus-1 (HIV-1) occurs due to HIV-1 rebound, the interruption of combination antiretroviral therapy, or development of drug resistance. Thus, novel HIV-1 inhibitors, targeting HIV-1 transcription are needed. We previously developed an HIV-1 transcription inhibitor, 1E7-03, that binds to the noncatalytic RVxF-accommodating site of protein phosphatase 1 and inhibits HIV-1 replication in cultured cells and HIV-1–infected humanized mice by impeding protein phosphatase 1 interaction with HIV-1 Tat protein. However, host proteins and regulatory pathways targeted by 1E7-03 that contribute to its overall HIV-1 inhibitory activity remain to be identified. To address this issue, we performed label-free quantitative proteome and phosphoproteome analyses of noninfected and HIV-1–infected CEM T cells that were untreated or treated with 1E7-03. 1E7-03 significantly reprogramed the phosphorylation profile of proteins including PPARα/RXRα, TGF-β, and PKR pathways. Phosphorylation of nucleophosmin (NPM1) at Ser-125 residue in PPARα/RXRα pathway was significantly reduced (>20-fold, *p* = 1.37 × 10^−9^), followed by the reduced phosphorylation of transforming growth factor-beta 2 at Ser-46 (TGF-β2, >12-fold, *p* = 1.37 × 10^−3^). Downregulation of NPM1’s Ser-125 phosphorylation was further confirmed using Western blot. Phosphorylation mimicking NPM1 S125D mutant activated Tat-induced HIV-1 transcription and exhibited enhanced NPM1–Tat interaction compared to NPM1 S125A mutant. Inhibition of Aurora A or Aurora B kinases that phosphorylate NPM1 on Ser-125 residue inhibited HIV-1, further supporting the role of NPM1 in HIV-1 infection. Taken together, 1E7-03 reprogrammed PPARα/RXRα and TGF-β pathways that contribute to the inhibition of HIV-1 transcription. Our findings suggest that NPM1 phosphorylation is a plausible target for HIV-1 transcription inhibition.

## Highlights

• 1E7-03, PP1 and HIV-1 transcription inhibitor, induces changes in cellular phosphorylation.
• 1E7-03 reprograms PPARα/RXRα, TGF-β and PKR pathways.
• 1E7-03 decreases phosphorylation of nucleophosmin (NPM1) at Ser-125 residue.
• Phosphorylation of NPM1 activates Tat-induced HIV-1 transcription.
• Inhibition of NPM1 upstream kinases, Aurora A or Aurora B, inhibites HIV-1.

## Graphical Abstract

**Figure:**
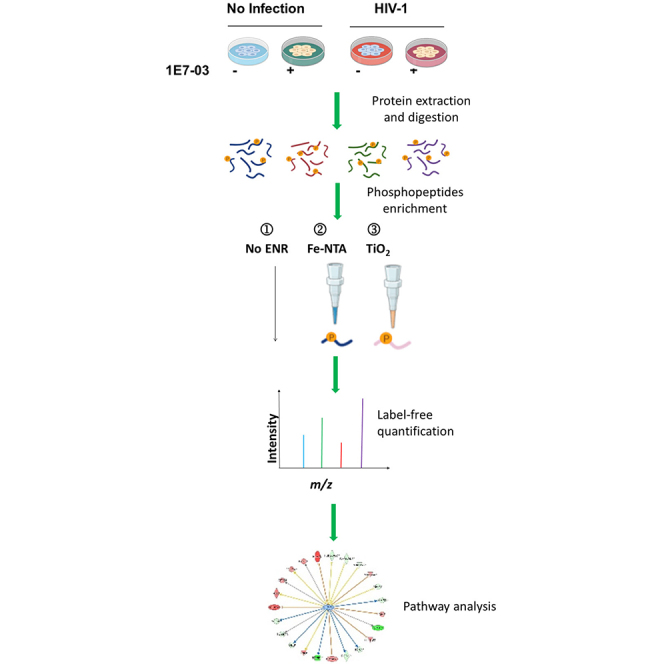

## In Brief

Small molecule HIV-1 transcription inhibitor, 1E7-03, that binds to the noncatalytic RVxF accommodating site of protein phosphatase 1 reprogramed protein phosphorylation in the PPARα/RXRα, TGF-β, and PKR pathways. Phosphorylation of nucleophosmin (NPM1) at Ser-125 residue was significantly reduced. NPM1 S125D mutant activated Tat-induced HIV-1 transcription and exhibited enhanced NPM1–HIV-1 Tat interaction. Inhibition of Aurora A or Aurora B kinases that phosphorylate NPM1 inhibited HIV-1. Our findings suggest that NPM1 phosphorylation is a plausible target for HIV-1 transcription inhibition.

Human immunodeficiency virus type 1 (HIV-1) remains a global public health threat as it escapes combination antiretroviral therapy (cART) by establishing long-lived stable reservoirs in quiescent memory T cells, tissue-resident macrophages, and hematopoietic stem cells (1, 2, 3). HIV-1 reservoirs can be reactivated when cART is interrupted or HIV-1 becomes resistant to cART regiments (4, 5). HIV-1 hijacks host proteins to optimize its replication and establish latency (6, 7). Transcriptional activation is an essential step in the reactivation of latent HIV-1 provirus and includes transcription chromatin remodeling, polymerase recruitment, transcription initiation, and elongation (3, 8, 9). Phosphorylation, a major posttranslational modification, functions as reversible molecular switch, controlling HIV-1 transcription and affecting formation of HIV-1 transcription elongation complexes (10, 11, 12, 13).

Protein phosphatase 1 (PP1) is a serine-threonine (Ser/Thr) phosphatase (14) composed of a catalytic subunit (PP1α, PP1β, or PP1γ) associated with one or two regulatory subunits that define its cellular localization, catalytic activity, and substrate-specificity (15). Vertebrates express over 200 PP1 regulatory subunits (16), which typically contain several PP1-binding motifs including RVxF, SpiDoc, SILK, MyPhoNE, ΦΦ, and NIPP1-helix (17, 18). Our previous study showed that HIV-1 Tat, a key activator of HIV-1 transcription, contains an “RVxF”-like motif (Q^35^VCF^38^) that interacts with PP1 and helps to translocate PP1 to the nucleus (19). The intrinsically weak Tat–PP1 interaction (Kd ∼ 1–10 μM) (19) can be targeted by therapeutic small molecules. Consecutively, we have developed several small molecules that targeted “RVxF”-accommodating cavity of PP1, disrupted the interaction between PP1 and Tat, and inhibited HIV-1 transcription (20, 21, 22, 23). 1E7-03 was identified as an effective HIV-1 transcription inhibitor, having unique 2,3-dihydro-1H-cyclopenta[*b*]quinoline scaffold that differs from FDA-approved anti–HIV-1 drugs and known HIV-1 transcription inhibitors (24). 1E7-03 inhibits HIV-1 replication in CEM T cells (IC_50_ ∼ 5 μM) with no toxicity (CC_50_ ∼ 100 μM) (22). Moreover, 1E7-03 significantly reduces HIV-1 mRNA production (∼40-fold reduction) in HIV-1–infected humanized mice (23). Recently, we confirmed that 1E7-03 binds directly to the “RVxF”-accommodating cavity of PP1 using ‘protein painting’ methodology along with the molecular docking and split NanoBit assay (25). However, host cellular factors modulated by PP1 and affected by 1E7-03 and their effect on HIV-1 transcription remain to be elucidated, especially in relation to the known host factors involved in HIV-1 transactivation regulation.

Previous quantitative phosphoproteome analyses focused on the phosphorylation of host proteins modulated during HIV-1 infection (26, 27). Here, we profiled global protein phosphorylation changes in CEM T cells induced by 1E7-03. Proteins potentially involved in HIV-1 transcription activation were identified using quantitative phosphoproteome and proteome analyses. The top targets were further validated using immunoblotting, site directed mutagenesis, and the use of specific kinase inhibitors.

## Experimental Procedures

### Experimental Design and Statistical Rationale

Noninfected CEM T cells untreated or treated with 1E7-03 were compared to CEM T cells infected with VSV-G–pseudotyped HIV-1 without or with 1E7-03 treatment. The sample size for each condition n = 1. Each sample was divided to three parts for phosphoenrichment. N = 12 samples were processed in instrumental triplicates and that gave N = 36 individual files to analyze. Trypsin-digested protein peptides, either nonenriched or enriched on Fe-NTA or TiO_2_ columns, were analyzed by tandem liquid chromatography-Fourier transform mass spectrometry (LC-FT/MS) followed by label-free quantitative analysis and Ingenuity Pathway Analysis (IPA). Biological functions and biological pathways were accessed by IPA with cutoff >1.5 fold and *p*-value <0.05.

### Chemicals

1E7-03 (purity above 98%) was synthesized by Enamine, as previously described (22). 1E7-03 was dissolved in dimethyl sulfoxide (DMSO) to obtain 10 mM stock solutions and stored at −20 °C. Acetonitrile and water-containing 0.1% formic acid (FA) were Optima LC/MS grade (Thermo Fisher Scientific). High-purity nitrogen (99.9%) was purchased from Roberts Oxygen Co, Inc. Other reagents were of analytical grade. DMSO and acetone were from Thermo Fisher Scientific.

### Plasmids

HIV-1 long terminal repeat–luciferase vector was kindly provided by Dr Manuel López-Cabrera (Unidad de Biología Molecular). FLAG-Tat expression vector was a gift from Dr Patricio Ray (Children’s National Research Institute). Expression vectors for GFP-nucleophosmin (NPM1) WT, GFP-NPM1 S125A, and GFP-NPM1 S125D were purchased from GenScript.

### Cell Culture, HIV-1 Infection, and 1E7-03 Treatment

CD4^+^ T cells (CEM T) and 293T cells were purchased from American Type Culture Collection. CEM T cells were cultured in RPMI media (Invitrogen) containing 10% fetal bovine serum (FBS) and 1% antibiotic solution (penicillin and streptomycin) at 37 °C and 5% CO_2_. For noninfected group, cells were seeded in 100 mm culture plates (2 × 10^5^ cells/ml) containing 10 ml of RPMI media and treated with 10 μM 1E7-03 or vehicle (DMSO) for 24 h. For HIV-1–infected group, CEM T cells were cultured in 100 ml flask (∼6 × 10^5^ cells/ml) and infected with VSVG pseudotyped pNL4-3.Luc.R-E-virus (HIV-1-LUC-G) at MOI = 0.01 for 24 h. Then, HIV-1–infected cells were seeded in 100 mm culture plates (2 × 10^5^ cells/ml) containing 10 ml of RPMI media and treated with 10 μM 1E7-03 or DMSO for additional 24 h. The 293T cells were cultured in Dulbecco’s modified Eagle’s medium (Invitrogen) containing 10% FBS and 1% antibiotic solution (penicillin and streptomycin).

### Cell Lysis and Protein Digestion

Cells were collected and washed three times with PBS. To obtain cell lysates, the cells were suspended in 1 ml cold whole cell lysis buffer (50 mM Tris–HCl, pH 7.5, 0.5 M NaCl, 1% NP-40, 0.1% SDS), supplemented with protease inhibitors (P8340-1 ML, Sigma Aldrich) and phosphatase inhibitors (P044-1 ML, Sigma Aldrich). The cytosolic protein fraction was isolated by centrifugation at 13,000*g* for 30 min at 4 °C to remove cellular debris. Protein concentration was determined using Bradford assay (Bio-Rad). An aliquot of 500 μg proteins from each sample was mixed with 4-fold volume cold acetone to precipitate proteins. All samples were centrifuged at 13,000*g* for 5 min. Precipitated proteins were collected and dried in SpeedVac concentrator. The pellet was resuspended in 500 μl of sodium phosphate buffer (pH 8.0), reduced in 10 mM DTT (1 h at 60 °C), alkylated with 30 mM iodoacetamide (20 min, room temperature in the dark), and digested with 10 μg trypsin (Promega) at 37 °C on orbital shaker overnight.

### Phosphopeptide Enrichment and Purification

An aliquot of 150 μl tryptic solution was processed by three different methods following manufacturer’s instructions. In nonenriched group (No ENR), peptide mixture was directly purified by Pierce Graphite Spin Columns (88302, Thermo Fisher Scientific). Briefly, columns were prepared by washing with 100 μl of 1 M NH_4_OH (×2), activating with 100 μl of acetonitrile, followed by 100 μl of 1% TFA washing (×2). Peptide mixtures were loaded into columns and incubated for 10 min with periodic vortex mixing. After incubation, each sample was cleaned by 200 μl of 1.0% TFA (×2) and eluted with 100 μl of 0.1% FA in 50% acetonitrile (×3). The elution was gently dried using a SpeedVac concentrator. In the Fe-NTA–enriched group, peptide mixtures were dried, resuspended in 200 μl of binding buffer, added to Fe-NTA spin columns (88300, Thermo Fisher Scientific), and incubated for 20 min at room temperature with end-over-end rotation. Samples were in turn washed with 100 μl of wash buffer A (×2), wash buffer B (×2), and ultrapure water. Then, samples were eluted with 50 μl of elution buffer (3–5 min incubation each time, ×2). The elution fractions were pooled together, acidified by adding 200 μl of 2.5% TFA, and purified using Graphite Spin Columns as described above. In TiO_2_-enriched group, TiO_2_ columns were prepared by washing with 20 μl of 0.4% TFA in 80% acetonitrile and 20 μl of 25% lactic acid. Peptide mixtures were dried, resuspended in 150 μl of 25% lactic acid, added to TiO_2_ spin columns (88301, Thermo Scientific), and incubated for 10 min at room temperature with end-over-end rotation (×2). Samples were washed with 20 μl of 25% lactic acid and 20 μl of 25% lactic acid (×2). Then, samples were eluted using 50 μl of 1.5% ammonium hydroxide and 50 μl of 5% pyrrolidine. The elution fractions were combined, acidified by adding 100 μl of 2.5% TFA, and purified using Graphite Spin Columns. Note: (×number) is repeated times of washing and spin speed for centrifuge for each step, please follow manufacturer’s instructions. We used one biological sample for each condition (four), which was processed using three different methods. Twelve samples were analyzed by mass spectrometry, using instrumental triplicates (in total, 36 samples).

### Nano LC-FT/MS Analysis

LC-FT/MS analysis was performed on a LC-20AD nano HPLC system (Shimadzu Corporation) coupled to Linear Trap Quadrupole (LTQ) XL Orbitrap mass spectrometer (Thermo Fisher Scientific) with the installed Xcalibur software (version 2.0.7, Thermo Fisher Scientific, https://www.thermofisher.com/order/catalog/product/OPTON-30965). Enriched and/or purified peptides were resuspended in 50 μl of water with 0.1% FA (v/v). A total of 10 μl of sample was loaded and washed for 6 min on a C_18_-packed precolumn (1 cm × 150 μm, 5 μm, 200 Å, Michrom Bioresources) with a solvent of A:B = 99:1 (A, 0.1% FA aqueous solution; B, 0.1% FA acetonitrile solution) at a constant flow of 12 μl/min. Peptides were transferred onward to an in house C_18_-packed analytical column (25 cm × 150 μm, 5 μm, 200 Å, Michrom Bioresources) and separated with a linear gradient of 6–55 min, 2–40% B; 55–62 min, 40–80% B; 62–70 min, 80% B (v/v) at the flow rate of 600 nl/min. Mobile phase A was 0.1% FA in water and mobile phase B was 0.1% FA in acetonitrile. The Orbitrap was operated under data-dependent acquisition mode. The spray voltage, capillary temperature, and capillary voltage were set to 2.0 kV, 200 °C, and 39.5 V, respectively. Full-scan mass spectra were acquired in Orbitrap over 300-2000 *m/z* with a resolution of 30,000, followed by MS^n^ scans by collision-induced dissociation activation mode. The three most intense ions were selected for fragmentation using collision-induced dissociation in the LTQ (normalized collision energy of 35, parent mass selection window of 2.5 Da, activation time of 30 ms, and minimum signal threshold for MS/MS scans set to 500 counts). Charge state rejection (charge state 1 was rejected) as well as dynamic exclusion (repeat counts, 2; repeat duration, 10 s; exclusion duration, 10 s) was enabled.

### Phosphoproteomic Data Analysis

LC-FT/MS raw data were searched by Proteome Discoverer 1.4 (PD 1.4) using SEQUEST search engine (Thermo Fisher Scientific), against the Uniprot Human database (11/26/2019, 140705 sequences) at a false discovery cut off ≤1%. A maximum of two missed cleavage sites was allowed with trypsin full cleavage. The mass tolerance for the precursor ion was 30 ppm and for the fragment - 0.1 Da. Phosphorylation of serine, threonine, and tyrosine residues were enabled as dynamic modifications, while carbamidomethylation of cysteine was set as fixed modification. Filters were set for peptides with different charges as follows: charge 2 = 1.5 (XCorr score), charge 3 = 2.0, and charges >4 =2.5 for highly confident peptides; charge 2 = 0.5, charge 3 = 0.8, and charges >4 =1 for modestly confident peptides. The label-free quantification of phosphopeptides eluting between 10 min and 70 min was performed with SIEVE 2.1 software (Thermo Fisher Scientific, https://tools.thermofisher.com/content/sfs/manuals/Man-XCALI-97695-SIEVE-22-Proteomics-ManXCALI97695-A-EN.pdf). Briefly, the chromatographic peaks detected by Orbitrap were aligned and the peptides peaks were detected with a minimum signal intensity of 1 × 10^5^; quantitative frames were determined based on *m/z* (width: 10 ppm) and retention time (width: 2.5 min). The PD 1.4.–identified phosphopeptides were uploaded as framing seeds. Statistical filters were set to assess the quality of the data. The CV raw MS intensities of the triplicates had to be within 30%. This helped minimizing the effect of run-to-run variability. Nonphosphorylated peptides were filtered out to eliminate their interference on the quantitative result. SIEVE 2.1 generated lists of differentially expressed proteins with a cut off >1.5-fold or <0.66 and *p*-value<0.05. The raw files and PD1. 4 search files can be accessed at https://massive.ucsd.edu/, The dataset access number is MSV000090850. The public access password is nekhai@1. The download ftp link is ftp://massive.ucsd.edu/MSV000090850/.

### Biological Function and Pathway Analysis

SIEVE 2.1 generated lists of differential phosphoproteins or expressed proteins with >1.5-fold of upregulation or downregulation and *p*-value<0.05 were uploaded to IPA (Ingenuity Systems) server for a Core analysis to investigate the protein function and biological networks. Canonical pathway analysis was carried out. PPARα/RXRα activation and TGF-β signaling networks were shown using Cytoscape 3.8.0.

### NanoBiT Assay

LgBiT-fused PRKA2A and SmBiT-fused PRKACA were obtained from Promega. 293T cells were plated in 96-well white/clear culture plates with 40% confluence and transiently transfected with the indicated constructs (1:1 ratio of interacting pairs) using Lipofectamine 3000 Plus in OPTI-MEM as directed by the manufacturer’s protocol. At 24 h post transfection, cells were treated with serial concentrations of 1E7-03 for the indicated amount of time. Nano-Glo Live Cell Substrate (N2012, Promega) was added, and luminescence was measured using a GloMax-Multi Detection System (Promega).

### Western Blot Analysis

CEM T cells or HEK293T cells expressing NPM1-EGFP were treated with 10 μM 1E7-03 or DMSO for 3 h or 24 h as indicated. The cells were also treated where indicated with 3 nM or 100 nM okadaic acid (OA) for 2 h. Total proteins were extracted using the whole cell lysis buffer supplemented with protease and phosphatase inhibitors (described above). Equal amounts of total protein were separated by electrophoresis and transferred to polyvinylidene fluoride membrane. The membrane was probed with antibodies for NPM1 (sc-529252, Santa Cruz Biotechnology, Inc), anti–p-S125 NPM1 antibodies (ab109546, Abcam, 1:1000 dilution), or GFP antibodies (ab290, Abcam, 1:1000 dilution).

### Transient Transfections

HEK293T cells were cultured in 24-well plates in Dulbecco’s modified Eagle’s medium containing 10% FBS. Cells were transfected at 30% confluence with Lipofectamine 3000 (Invitrogen), according to the manufacturer’s recommendations. Cells were cultured 48 h post-transfection, and luciferase activity was analyzed using Luclite plus Reporter Gene Assay (PerkinElmer) measured by Glo-Max Microplate Multimode reader (Promega).

### Coimmunoprecipitation

HEK293T cells were transfected with the indicated FLAG-Tat and GFP-NPM1 vectors, as described above. The whole cell extracts were prepared using the whole cell lysis buffer supplemented with protease and phosphatase inhibitors (described above). About 300 μg of whole cell extract supplemented with 8 μg of anti-FLAG antibodies was incubated with 5% bovine serum albumin preblocked protein A/G-agarose beads in TNN buffer (50 mM Tris–HCl, pH 7.5, 0.5% NP-40, 150 mM NaCl) at room temperature for 4 h with rocking. The agarose beads were precipitated and washed with TNN buffer. Proteins were resolved on 10% Bis-Tris SDS-PAGE, transferred to polyvinylidene fluoride membrane, and probed with the indicated antibodies.

### Colocalization Analysis

HEK293T cells were transfected with vectors expressing EGFP-tagged Tat and RFP-tagged NPM1, WT, and mutants. At 24 h post transfection, the cells were photographed on Olympus IX51 using a filter for FITC and Texas Red fluorescence at 400× magnification. Quantification of the colocalization was conducted in Image J using JACoP plug-in that allows Pearson’s correlation analysis. The image colors were split, and threshold parameters were adjusted prior to the correlation analysis.

### One round HIV-1 Infection Assay

CEM T cells were infected with HIV-1-LUC-G and cultured in 96-well white plates (3 × 10^5^ cells/ml, 100 μl/well) at 37 °C and 5% CO_2_. The cells were treated with serial dilutions of 1E7-03, Aurora A inhibitor, Barasertib (Aurora B inhibitor), or vehicle (DMSO). At 24 h posttreatment, 100 μl of reconstituted luciferase buffer (Luclite Kit, PerkinElmer) was added to each well, incubated for 10 min, and luminescence was measured using Glo-Max Microplate Multimode reader (Promega).

### Cell Viability Assays

Cell viability was measured with MTT assay. CEM T cells (3 × 10^5^) were grown in 96-well plates and treated overnight with serially diluted compounds or DMSO control. Post treatment, 10 μl of MTT solution (ATCC 30–1010K) was added to each well, and the samples were incubated at 37 °C for 2 h. Then, 100 μl of lysis buffer (0.03% HCl with 10% SDS) was added to each well. The cells were incubated for 20 min in the dark. Absorbance was measured at 490 nm at a microplate reader (Bio-Rad Model 680). Each point was measured in triplicate, and serum-free medium with MTT solution was used as a negative control.

### Fluorescence Assisted Cell Sorting

CEM T cells (1 × 10^6^ cells) were fixed for 5 min at room temperature in 4% paraformaldehyde and then permeabilized for 15 min in cytofix/cytoperm buffer (cat. #554714, BD Pharmingen). Cells were stained with p24-PE antibodies (cat# 6604667, HIV-1 core antigen-RD1, KC57, Beckman Coulter Life Sciences) for 1 h at 4 °C in the dark. After staining, the cells were washed and analyzed in BD FACS Calibur (BD Biosciences) using FlowJo software (https://www.bdbiosciences.com/en-us/products/software/flowjo-v10-software).

### Statistical Analysis

All graphs were prepared using GraphPad prism 6 software. Data are presented as mean ± SD or SEM as indicated in the figure legends. Means were compared with Student *t*-tests.

## Results

### Phosphopeptide Enrichment

**Fig. 1:**
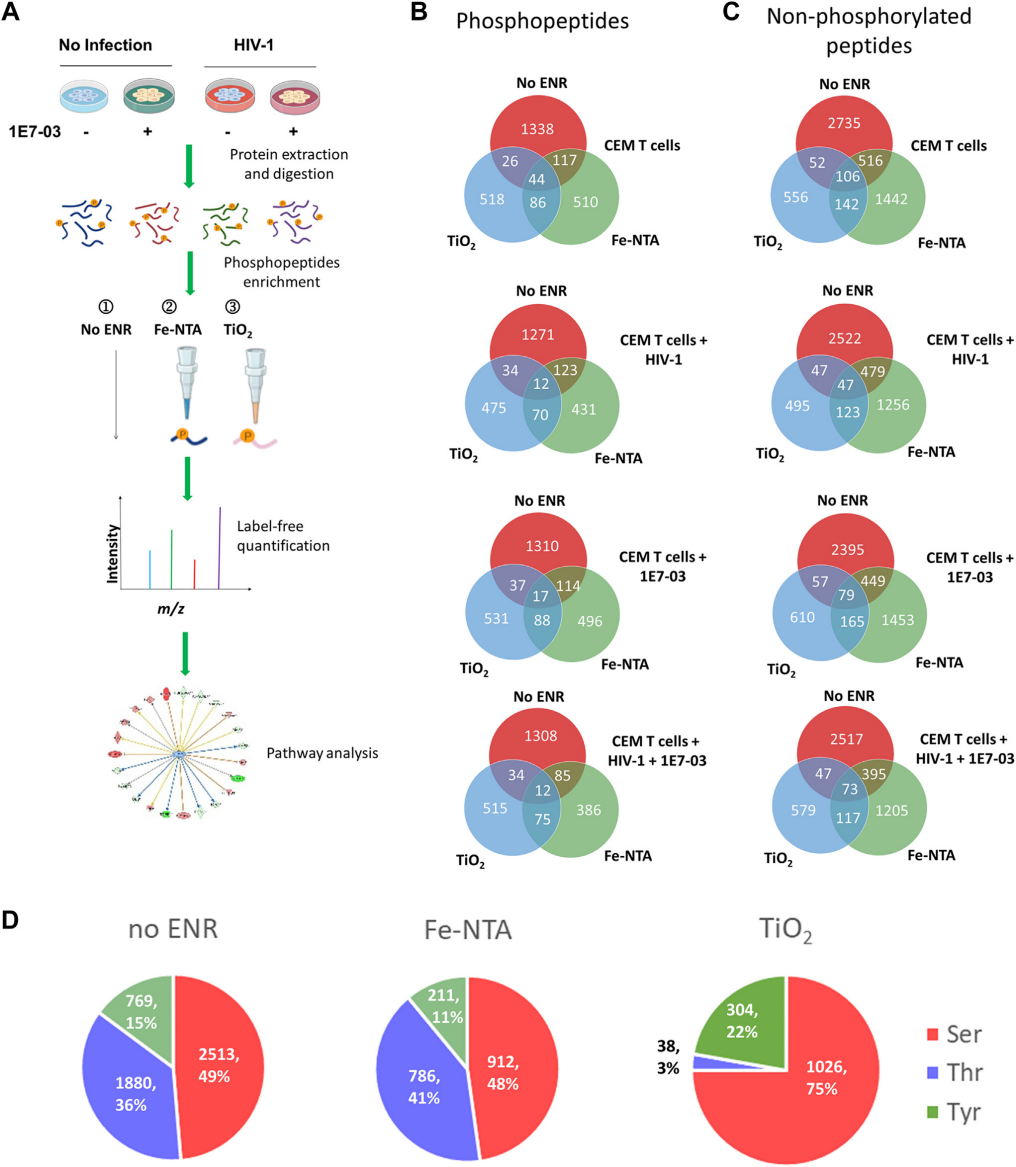
**Experimental design and phosphopeptide enrichment.***A*, workflow for label-free quantitative phosphoproteomics. Noninfected and HIV-1–infected CEM T cells were treated with 10 μM 1E7-03 or DMSO control for 24 h. Total protein was extracted, digested with trypsin, and either directly purified by Pierce Graphite Spin Columns without enrichment (No ENR) or enriched by Fe-NTA or TiO_2_ phosphopeptide enrichment kits prior to the spin column purification. Peptides were analyzed by LC-FT/MS and quantified using label-free workflow. Biological function and networks within the quantified differentially phosphorylated proteins were determined by IPA. *B*, comparison of nonenriched, Fe-NTA, and TiO2-enriched phosphopeptides. *C*, comparison of nonphosphorylated peptides in nonenriched, Fe-NTA, and TiO2-enriched sets. *D*, serine (Ser), threonine (Thr), and tyrosine (Tyr)-phosphorylated peptides identified in nonenriched, Fe-NTA, and TiO_2_-enriched samples. IPA, ingenuity pathway analysis; HIV-1, human immunodeficiency virus-1; LC-FT/MS, tandem liquid chromatography-Fourier transform mass spectrometry; DMSO, dimethyl sulfoxide.

**Fig. S1:**
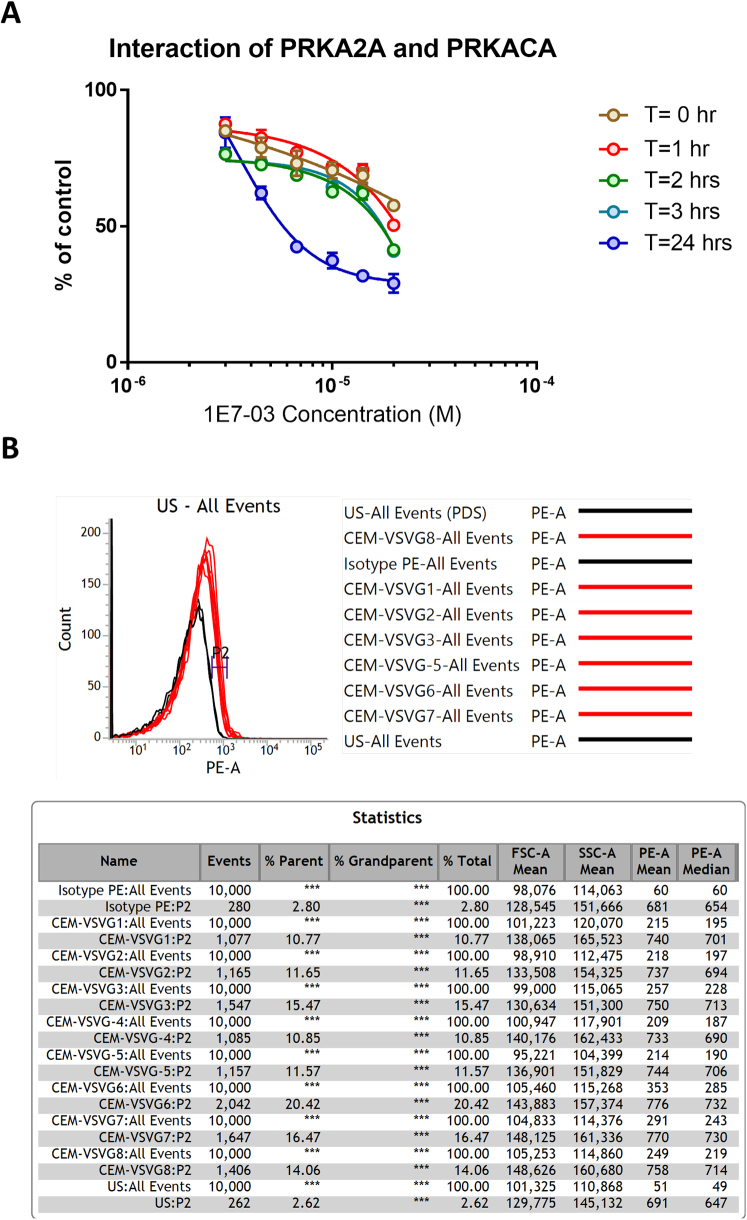
*A*, Effect of 1E7-03 on the interaction of PRKA2A and PRKACA. The interaction was measured by Split NanoBiT assay in HEK293T transfected with plasmids expressing small and large-bit fused PRKA2A and PRKACA for 24 h and treated with different concentrations of 1E7-03 for the indicated time. Data are shown as percent of the untreated control. Shown are the normalized mean ± SD of triplicates. *B*, FACS analysis of CEM T cells infected with VSVg-pseudotyped HIV-1. Infected CEM T cells were stained with p24-PE antibodies and analyzed in BD FACSCalibur. FlowJo software was used to plot overlayed histograms and derived statistics.

To gain an insight on the host proteins phosphorylation induced by 1E7-03, we compared noninfected CEM T cells, untreated or treated with 1E7-03 (Fig. 1*A*, no infection) and CEM T cells infected with VSV-G–pseudotyped HIV-1 virus expressing luciferase (HIV-1-LUC-G, MOI = 0.01) without or with 1E7-03 treatment for 24 h (Fig. 1*A*, HIV-1). The treatment time was chosen based on the effect of 1E7-03 on PP1-dependent interaction of PRKA2a and PRKACA which only showed the effect at 24 h and not at 3 h treatment time (supplemental Fig. S1*A*). HIV-1 infection efficiency determined by FACS analysis of CEM T cells stained with anti-p24 antibodies showed that about 14% of CEM T cells were infected (supplemental Fig. S1*B*). Trypsin-digested protein peptides without enrichment (Fig. 1*A*, no ENR) or enriched on Fe-NTA or TiO_2_ columns (Fig. 1*A*, Fe-NTA and TiO_2_) were analyzed by LC-FT/MS followed by label-free quantitative analysis and IPA (Fig. 1*A*). We detected peptides phosphorylated on serine and threonine residues and included in the analysis peptides phosphorylated on tyrosine. We observed approximately 1500, 700, and 600 unique phosphopeptides in the nonenriched, TiO_2_-, and Fe-NTA–enriched individual groups, respectively (Fig. 1*B*, see combined peptides in supplemental Table S1 and Proteome Discoverer 1.4 for identified proteins in supplemental Table S2). We also detected approximately 3500, 800, and 2200 nonphosphorylated peptides in the nonenriched, TiO_2_-, and Fe-NTA–enriched individual groups (Fig. 1*C*). Number of specific serine- and threonine-phosphorylated residues were equal in the nonenriched and Fe-NTA–enriched peptide groups, whereas TiO_2_-enriched peptide group contained primarily serine phosphorylated peptides (Fig. 1*D*).

### Phosphorylation Profile of Host Proteins Reprogramed by 1E7-03

**Fig. 2:**
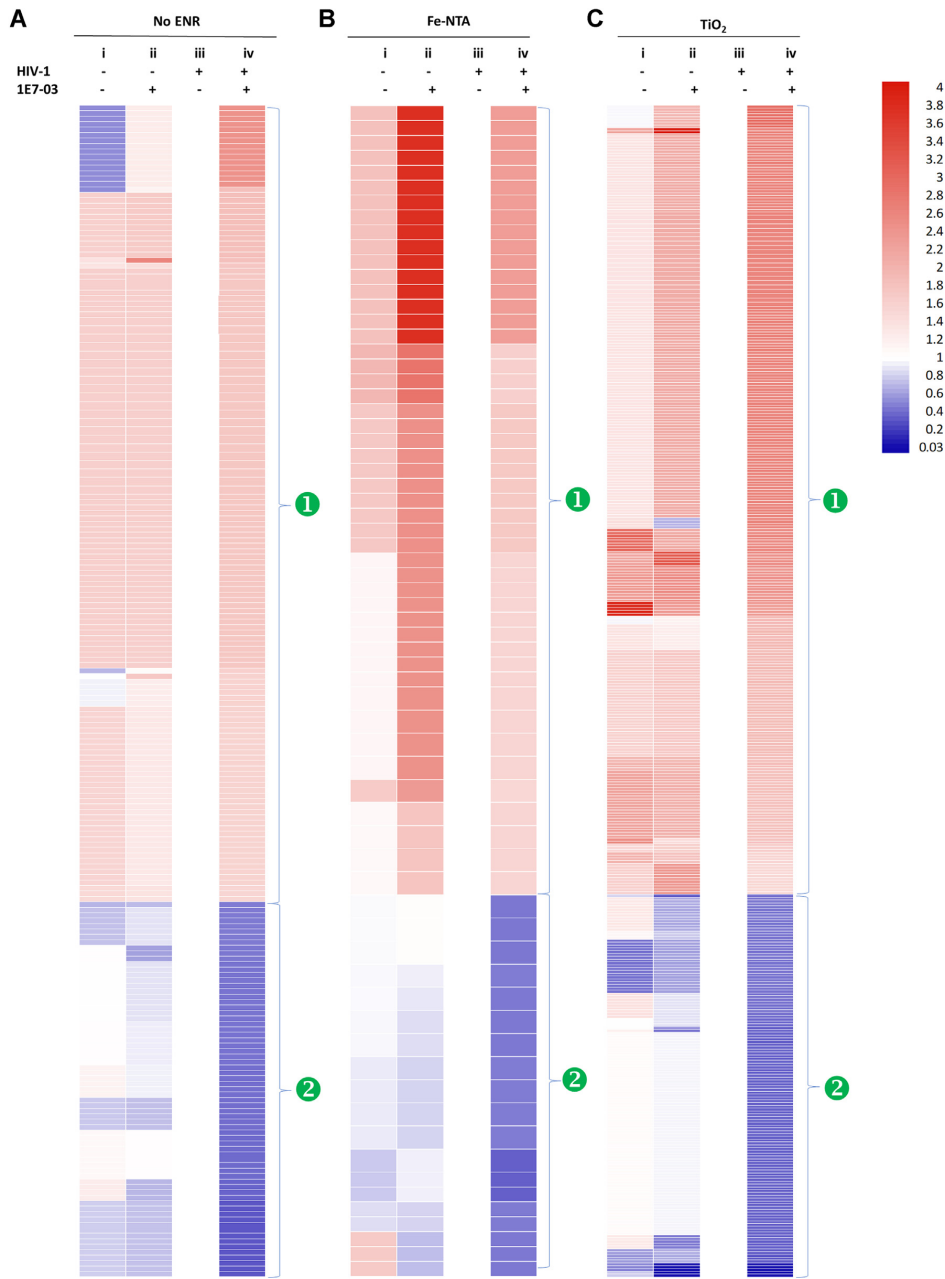
**Heatmap analysis of host phosphoproteins affected by 1E7-03.** Noninfected cells without or with 1E7-03 treatment (groups i and ii) and HIV-1–infected CEM T cells without or with 1E7-03 treatment (groups iii and iv) were compared using group iii as a reference. Fold change>1.5 and *p* < 0.05 were used as a cutoff. Annotated are proteins whose phosphorylation was increased (❶) or decreased (❷) by 1E7-03. *A*, proteins in the nonenriched groups. *B*, proteins in Fe-NTA–enriched groups. *C*, proteins in TiO_2_-enriched groups. HIV-1, human immunodeficiency virus-1.

CEM T cells, noninfected and untreated or treated with 1E7-03 (Fig. 2, groups i and ii) and HIV-1–infected CEM T cells treated with 1E7-03 (Fig. 2, group iv) were compared to CEM T cells infected with HIV-1 and not treated with 1E7-03 that was designated as a reference group in SIEVE 2.1 label-free quantification analysis (cutoff >1.5-fold and *p* value<0.05) (Fig. 2, group iii). SIEVE 2.1 ion integration data are shown in supplemental Tables S3–S8 and summarized in supplemental Table S9. The overall phosphorylation patterns induced by 1E7-03 treatment, that we observed, fell into two categories: 1) increased phosphorylation by 1E7-03 and 2) decreased phosphorylation by 1E7-03 when groups i and ii, and groups iii and iv were compared (Fig. 2). In Fe-NTA–enriched groups (Fig. 2*B* and supplemental Table S9), 1E7-03 increased protein phosphorylation to higher levels in noninfected cells than HIV-1–infected cells, whereas 1E7-03 reduced protein phosphorylation in HIV-1–infected cells to higher extent than the noninfected cells. In nonenriched and TiO_2_-enriched groups (Fig. 2, *A* and *C*, and supplemental Table S9), 1E7-03 similarly increased and decreased protein phosphorylation in HIV-1 infected, whereas in the noninfected cells, many peptides remain phosphorylated regardless of 1E7-03 treatment. Despite only 14% of the cells being infected, 1E7-03 induced more significant phosphorylation changes in HIV-1–infected cells comparing to non-infected cells suggesting potential interactions between the host, the virus, and the drug.

### 1E7-03 Induces Phosphorylation Changes in PPARα/RXRα and TGF-β Pathways

**Fig. 3:**
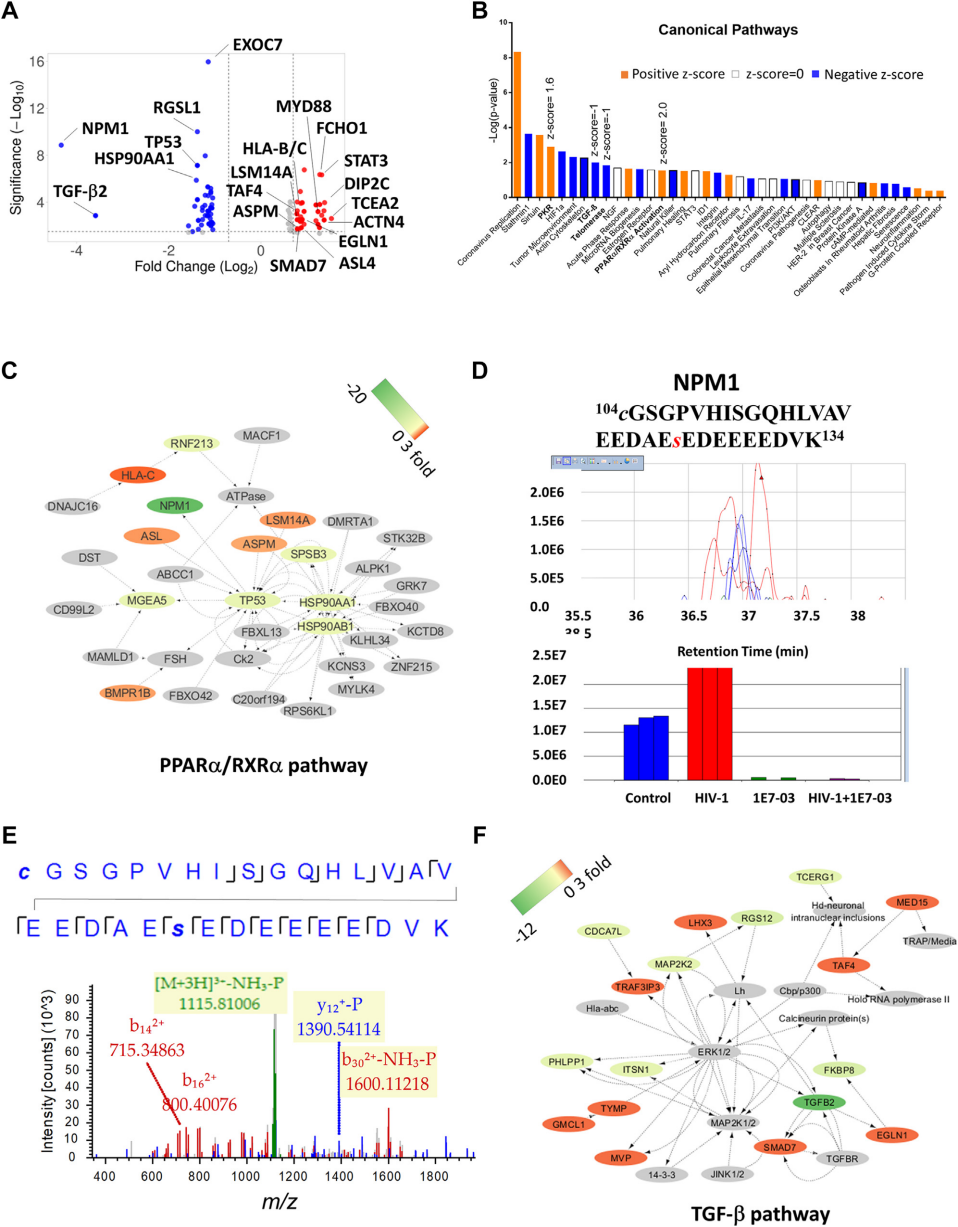
**Canonical pathway analysis of host phosphoproteins targeted by 1E7-03.***A*, volcano plot shows host protein phosphorylation significantly changed by 1E7-03 with cutoff >1.5-fold and *p* < 0.05 (*red* color - upregulation; *blue* color - downregulation). Labeled are proteins participating in HIV-1 replication. *B*, differentially phosphorylated proteins were analyzed by Ingenuity Pathway Analysis (IPA) for canonical pathways. Colors indicate pathway activation with positive z-score (*orange*); no activation with z-score=0 (*white*); and pathway downregulation with negative z-score (*blue*). *C*, PPARα/RXRα network changes induced by 1E7-03. *Orange* colors represent upregulation of phosphorylation, *green* colors represent downregulation, and *gray* color represents no data available. The change in phosphorylation scale of -20 to 3 is shown. *D*, label-free quantification analysis by SIEVE 2.1 for the phosphopeptide ^104^*c*GSGPVHISGQHLVAVEEDAEsEDEEEEDVK^134^ derived from NPM1. Ion elution profiles (*top*) and integrated intensities (*bottom*) generated by SIEVE 2.1 are shown in *blue* for noninfected cells, in *red* for HIV-1–infected cells, in *green* for noninfected cells plus 1E7-03 treatment, and in *purple* for HIV-1–infected cells plus 1E7-03 treatment. Data are shown in triplicates. *E*, representative MS/MS spectrum of the phosphopeptide ^104^*c*GSGPVHISGQHLVAVEEDAEsEDEEEEDVK^134^ derived from NPM1. The colored peaks indicate matched MS/MS fragments. *Green* color indicates precursor ion; *blue* and *red* colors indicate y and b ions, respectively. *F*, TGF-β–signaling network changes induced by 1E7-03. *Orange* colors represent upregulation of phosphorylation; *green* colors represent downregulation; and *gray* color represents no quantitative data available. The change in phosphorylation scale of -12 to 3 is shown. HIV-1, human immunodeficiency virus-1; NPM1, nucleophosmin.

Differentially phosphorylated proteins quantified by SIEVE 2.1 were further analyzed by IPA software that allowed data consolidation and removal of the multiplications. We combined results from nonenriched and Fe-NTA– and TiO2-enriched groups with HIV-1 infection to identify proteins whose phosphorylation was upregulated and downregulated after 1E7-03 treatment (Fig. 3A, Tables 1 and S9). 1E7-03 significantly upregulated phosphorylation of 541 proteins including ACTN4 (2.59-fold increase, *p =* 3.23 × 10^-3^), ACPM (1.6-fold increase, *p =* 0.01), HLA-B/C (2.59-fold increase, *p* = 3.23 × 10^−3^), EGLN1 (1.86-fold increase, *p* = 0.003), ASL (1.84-fold increase, *p* = 0.015), LSM14A (1.83-fold increase, *p* = 4.31 × 10^−3^), SMAD7 (1.73-fold increase, *p* = 0.018), TAF4 (1.73-fold increase, *p* = 5.3 × 10^−4^), and several additional proteins (Fig. 3*A*, Tables 1 and S9). Among 542 proteins whose phosphorylation was downregulated by 1E7-03, NPM1 showed the strongest effect (20.15-fold downregulation, *p* = 1.37 × 10^−9^), followed by TGF-β2 (12-fold decrease, *p* = 1.37 × 10^−3^), TP53 (p53) (2.6-fold decrease, *p* = 7.36 × 10^−8^), HSP90AA1 (2.6-fold decrease, *p* = 1.33 × 10^−6^), and HSP90AB1 (2.2-fold decrease, *p* = 4.93 × 10^−6^) (Fig. 3*A*, Tables 1 and S9). IPA was used to define biological pathways affected by 1E7-03. Canonical pathway analysis showed that 1E7-03 significantly upregulated PPARα/RXRα pathway (Z-score = 2.0) and PKR pathway (Z-score = 1.6) and downregulated transforming growth factor beta (TGF-β) (Z-score = −1) and telomerase (Z-score = −1) pathways (Fig. 3*B* and supplemental Table S10). Additional pathways affected by 1E7-03 are listed in the supplemental Table S10.

**Table 1:** Proteins whose phosphorylation was changed by 1E7-03 treatment

Symbol	Entrez gene name	Expr fold change	Expr *p*-value	Location	Type(s)
MACF1	Microtubule actin crosslinking factor 1	2.892	0.0338	Cytoplasm	Enzyme
TCEA2	Transcription elongation factor A2	2.892	0.00243	Nucleus	Transcription regulator
DIP2C	Disco-interacting protein 2 homolog C	2.609	0.000107	Other	Other
ABI2	Abl interactor 2	2.591	0.00323	Cytoplasm	Other
ACTN4	Actinin alpha 4	2.591	0.00323	Cytoplasm	Transcription regulator
DSG2	Desmoglein 2	2.591	0.00323	Plasma membrane	Other
FBXO42	F-box protein 42	2.591	0.00323	Other	Other
HLA-B	Major histocompatibility complex, class I, B	2.591	0.00323	Plasma membrane	Transmembrane receptor
HLA-C	Major histocompatibility complex, class I, C	2.591	0.00323	Plasma membrane	Other
MORN1	MORN repeat–containing 1	2.591	0.00323	Other	Other
PRELID3B	PRELI domain–containing 3B	2.591	0.00323	Cytoplasm	Other
SF3B1	Splicing factor 3b subunit 1	2.591	0.00323	Nucleus	Other
SRRM5	Serine/arginine repetitive matrix 5	2.591	0.00323	Other	Other
ZNF503-AS2	ZNF503 antisense RNA 2	2.591	0.00323	Other	Other
C2CD5	C2 calcium–dependent domain containing 5	2.584	0.000527	Cytoplasm	Other
SRSF11	Serine and arginine rich splicing factor 11	2.499	0.00128	Nucleus	Other
STAT3	Signal transducer and activator of transcription 3	2.499	4.63E-07	Nucleus	Transcription regulator
FCHO1	FCH and mu domain–containing endocytic adaptor 1	2.426	4.39E-07	Plasma membrane	Other
LRIT2	Leucine rich repeat, Ig-like, and transmembrane domains 2	2.426	0.000154	Other	Other
PNCK	Pregnancy upregulated nonubiquitous CaM kinase	2.426	0.000154	Other	Kinase
GRM2	Glutamate metabotropic receptor 2	2.415	0.0443	Plasma membrane	G-protein–coupled receptor
PLG	Plasminogen	2.415	0.0142	Extracellular space	Peptidase
PPP1R26	Protein phosphatase 1 regulatory subunit 26	2.415	0.0443	Nucleus	Other
SPATA33	Spermatogenesis-associated 33	2.415	0.0443	Other	Other
SPOUT1	SPOUT domain–containing methyltransferase 1	2.415	0.0142	Nucleus	Other
TRDV1	T cell receptor delta variable 1	2.415	0.0443	Other	Other
DMAC2	Distal membrane arm assembly complex 2	2.363	0.00762	Cytoplasm	Other
MYD88	MYD88 innate immune signal transduction adaptor	2.363	0.000624	Plasma membrane	Other
PCDHB14	Protocadherin beta 14	2.363	0.000624	Plasma membrane	Other
PER1	Period circadian regulator 1	2.363	0.00762	Nucleus	Transcription regulator
STX18	Syntaxin 18	2.363	0.00762	Cytoplasm	Transporter
TBC1D29P	TBC1 domain family member 29, pseudogene	2.363	0.00762	Other	Other
CCDC7	Coiled-coil domain–containing 7	2.292	0.00371	Other	Other
CWC22	CWC22 spliceosome-associated protein homolog	2.292	0.000168	Nucleus	Other
TRPS1	Transcriptional repressor GATA binding 1	2.292	0.000168	Nucleus	Transcription regulator
WDFY3	WD repeat and FYVE domain–containing 3	2.292	0.000168	Cytoplasm	Enzyme
SLC6A15	Solute carrier family 6 member 15	1.922	1.74E-07	Plasma membrane	Transporter
FLNA	Filamin A	1.916	0.000603	Cytoplasm	Other
GCC2	GRIP and coiled-coil domain–containing 2	1.916	0.00747	Cytoplasm	Other
LRRCC1	Leucine rich repeat and coiled-coil centrosomal protein 1	1.916	0.000603	Nucleus	Transporter
ZNF362	Zinc finger protein 362	1.916	0.00747	Other	Other
C1QL3	Complement C1q like 3	1.863	0.00294	Extracellular space	Other
C2orf16	Chromosome 2 open reading frame 16	1.863	0.00294	Other	Other
EGLN1	Egl-9 family hypoxia inducible factor 1	1.863	0.00294	Cytoplasm	Enzyme
FNDC3A	Fibronectin type III domain–containing 3A	1.863	0.00294	Cytoplasm	Other
GINS1	GINS complex subunit 1	1.863	0.00294	Nucleus	Other
MYO3A	Myosin IIIA	1.863	0.00294	Cytoplasm	Kinase
NAV3	Neuron navigator 3	1.863	0.000109	Nucleus	Other
NHLRC1	NHL repeat–containing E3 ubiquitin protein ligase 1	1.863	0.00294	Cytoplasm	Enzyme
ODF3	Outer dense fiber of sperm tails 3	1.863	0.00294	Cytoplasm	Other
OSBPL5	Oxysterol-binding protein like 5	1.863	0.000109	Cytoplasm	Transporter
TRIO	Trio Rho guanine nucleotide exchange factor	1.863	0.00294	Cytoplasm	Kinase
ZNF226	Zinc finger protein 226	1.863	0.00294	Nucleus	Transcription regulator
NEFH	Neurofilament heavy	1.846	0.0041	Cytoplasm	Other
ASL	Argininosuccinate lyase	1.837	0.0146	Cytoplasm	Enzyme
C8orf34	Chromosome 8 open reading frame 34	1.837	0.0146	Other	Other
EIF2D	Eukaryotic translation initiation factor 2D	1.837	0.0146	Cytoplasm	Transporter
GPRC5C	G protein–coupled receptor class C group 5 member C	1.837	0.0146	Plasma membrane	G-protein–coupled receptor
TUBA1C	Tubulin alpha 1c	1.837	0.0146	Cytoplasm	Other
CHD4	Chromodomain helicase DNA-binding protein 4	1.826	0.00311	Nucleus	Enzyme
CNTNAP5	Contactin-associated protein like 5	1.826	0.000122	Other	Other
DDX21	DExD-box helicase 21	1.826	0.00311	Nucleus	Enzyme
FNDC1	Fibronectin type III domain–containing 1	1.82	0.0017	Plasma membrane	Other
ARRDC2	Arrestin domain–containing 2	1.811	1.21E-05	Other	Other
FANCI	FA complementation group I	1.811	0.00431	Nucleus	Other
GMPPB	GDP-mannose pyrophosphorylase B	1.811	0.00431	Cytoplasm	Enzyme
LONP2	Lon peptidase 2, peroxisomal	1.811	0.00431	Cytoplasm	Peptidase
LSM14A	LSM14A mRNA processing body assembly factor	1.811	0.00431	Cytoplasm	Other
METTL1	Methyltransferase like 1	1.811	0.00431	Nucleus	Enzyme
PAH	Phenylalanine hydroxylase	1.811	0.00431	Cytoplasm	Enzyme
SGSH	N-sulfoglucosamine sulfohydrolase	1.811	0.00431	Cytoplasm	Enzyme
TRAF3IP3	TRAF3-interacting protein 3	1.811	0.00431	Other	Other
PBX4	PBX homeobox 4	1.773	0.00314	Nucleus	Transcription regulator
ZNF197	Zinc finger protein 197	1.773	0.0187	Nucleus	Transcription regulator
ZNF585B	Zinc finger protein 585B	1.736	0.0385	Nucleus	Other
ALG3	ALG3 alpha-1,3- mannosyltransferase	1.73	0.00304	Cytoplasm	Enzyme
ARHGAP26	Rho GTPase-activating protein 26	1.73	0.00304	Cytoplasm	Other
ARHGEF16	Rho guanine nucleotide exchange factor 16	1.73	0.0184	Cytoplasm	Other
BMPR1B	Bone morphogenetic protein receptor type 1B	1.73	0.0184	Plasma membrane	Kinase
C2orf42	Chromosome 2 open reading frame 42	1.73	0.0184	Nucleus	Other
COL6A2	Collagen type VI alpha 2 chain	1.73	0.0184	Extracellular space	Other
DOT1L	DOT1-like histone lysine methyltransferase	1.73	0.0184	Nucleus	Phosphatase
DRC7	Dynein regulatory complex subunit 7	1.73	0.0184	Cytoplasm	Other
M1AP	Meiosis 1–associated protein	1.73	0.0184	Cytoplasm	Other
MED15	Mediator complex subunit 15	1.73	0.0184	Nucleus	Transcription regulator
MTG2	Mitochondrial ribosome-associated gtpase 2	1.73	0.0184	Cytoplasm	Enzyme
NSMCE4A	NSE4 homolog A, SMC5-SMC6 complex component	1.73	0.0184	Nucleus	Other
PHYHD1	Phytanoyl-coa dioxygenase domain–containing 1	1.73	0.0184	Other	Other
POMT2	Protein O-mannosyltransferase 2	1.73	0.00304	Cytoplasm	Enzyme
QKI	QKI, KH domain–containing RNA binding	1.73	0.0184	Nucleus	Other
SCLY	Selenocysteine lyase	1.73	0.0184	Cytoplasm	Enzyme
SMAD7	SMAD family member 7	1.73	0.0184	Nucleus	Transcription regulator
TAF4	TATA-box–binding protein associated factor 4	1.73	0.000527	Nucleus	Transcription regulator
TUBA8	Tubulin alpha 8	1.73	0.00304	Cytoplasm	Other
TUBA1B	Tubulin alpha 1b	1.73	0.00304	Cytoplasm	Other
TUBA4A	Tubulin alpha 4a	1.73	0.00304	Cytoplasm	Other
TUBA4B	Tubulin alpha 4b	1.73	0.00304	Cytoplasm	Other
TYMP	Thymidine phosphorylase	1.73	0.0184	Extracellular space	Growth factor
UTY	Ubiquitously transcribed tetratricopeptide repeat–containing, Y-linked	1.73	9.42E-05	Nucleus	Enzyme
ZNF90	Zinc finger protein 90	1.73	0.0184	Nucleus	Transcription regulator
ZNF474	Zinc finger protein 474	1.73	0.00304	Other	Other
ZSCAN18	Zinc finger and SCAN domain–containing 18	1.73	0.0184	Other	Other
BAHCC1	BAH domain and coiled-coil–containing 1	1.719	0.000981	Other	Other
PFKFB3	6-phosphofructo-2-kinase/fructose-2,6-biphosphatase 3	1.719	0.000981	Cytoplasm	Kinase
TNRC18	Trinucleotide repeat–containing 18	1.719	0.000981	Nucleus	Other
OBSCN	Obscurin, cytoskeletal calmodulin, and titin-interacting rhogef	1.651	0.0346	Cytoplasm	Kinase
ASPM	Abnormal spindle microtubule assembly	1.608	0.0106	Nucleus	Other
LOC105370980	Putative uncharacterized protein UNQ9370/PRO34162	1.591	6.83E-08	Other	Other
ASMTL	Acetylserotonin O-methyltransferase like	1.585	0.0224	Cytoplasm	Enzyme
TRRAP	Transformation/transcription domain–associated protein	1.585	0.0224	Nucleus	Transcription regulator
ANKLE2	Ankyrin repeat and LEM domain–containing 2	1.566	0.0277	Nucleus	Transcription regulator
BTAF1	B-TFIID TATA-box–binding protein associated factor 1	1.566	0.0277	Nucleus	transcription regulator
LHX3	LIM homeobox 3	1.566	0.0277	Nucleus	transcription regulator
MAP3K4	Mitogen-activated protein kinase kinase kinase 4	1.566	0.0277	Cytoplasm	Kinase
PGLYRP4	Peptidoglycan recognition protein 4	1.566	0.00628	Plasma Membrane	Transmembrane receptor
POLQ	DNA polymerase theta	1.566	0.0277	Nucleus	Enzyme
PPAN	Peter pan homolog	1.566	0.00628	Nucleus	Other
PPAN-P2RY11	PPAN-P2RY11 readthrough	1.566	0.00628	Other	Other
PTPRS	Protein tyrosine phosphatase receptor type S	1.566	0.0277	Plasma Membrane	Phosphatase
TOGARAM1	TOG array regulator of axonemal microtubules 1	1.566	0.0277	Extracellular Space	Other
USP47	Ubiquitin-specific peptidase 47	1.566	0.0277	Cytoplasm	Peptidase
CD99L2	CD99 molecule like 2	1.554	0.00268	Plasma Membrane	Other
GMCL1	Germ cell-less 1, spermatogenesis associated	1.554	9.21E-05	Nucleus	Other
GMCL2	Germ cell-less 2, spermatogenesis associated	1.554	9.21E-05	Nucleus	Other
SYNE2	Spectrin repeat–containing nuclear envelope protein 2	1.553	0.000204	Nucleus	Other
CCDC171	Coiled-coil domain–containing 171	1.545	0.00176	Other	Other
PGK2	Phosphoglycerate kinase 2	1.543	0.000847	Cytoplasm	Kinase
SNX18	Sorting nexin 18	1.543	0.000847	Cytoplasm	Transporter
TTC21A	Tetratricopeptide repeat domain 21A	1.543	0.000847	Extracellular space	Other
BBS7	Bardet-Biedl syndrome 7	1.536	0.0183	Cytoplasm	Other
MAGED2	MAGE family member D2	1.51	0.000488	Plasma membrane	Other
MINAR1	Membrane integral NOTCH2-associated receptor 1	1.51	0.00665	Plasma membrane	Other
MVP	Major vault protein	1.51	0.00665	Nucleus	Other
PKDREJ	Polycystin family receptor for egg jelly	1.51	0.000488	Plasma membrane	Ion channel
SP140	SP140 nuclear body protein	1.51	0.00665	Nucleus	Transcription regulator
ZNF831	Zinc finger protein 831	1.51	0.00665	Other	Other
DELE1	DAP3-binding cell death enhancer 1	−2.058	0.00645	Cytoplasm	Other
OAS3	2′-5′-oligoadenylate synthetase 3	−2.06	0.0217	Cytoplasm	Enzyme
RTTN	Rotatin	−2.064	4.26E-05	Cytoplasm	Other
ERCC6L2	ERCC excision repair 6–like 2	−2.088	0.00521	Other	Enzyme
FAM187A	Family with sequence similarity 187 member A	−2.088	0.000313	Other	Other
GCN1	GCN1 activator of EIF2AK4	−2.088	0.00521	Cytoplasm	Translation regulator
RALGAPA1	Ral gtpase-activating protein catalytic alpha subunit 1	−2.088	0.000313	Cytoplasm	Other
RSC1A1	Regulator of solute carriers 1	−2.088	0.00521	Nucleus	Other
MAP2K2	Mitogen-activated protein kinase kinase 2	−2.09	0.000158	Cytoplasm	Kinase
ZNF99	Zinc finger protein 99	−2.107	0.00882	Other	Other
FMNL1	Formin-like 1	−2.122	0.00102	Cytoplasm	Other
MAP4	Microtubule-associated protein 4	−2.122	1.23E-05	Cytoplasm	Other
MDC1	Mediator of DNA damage checkpoint 1	−2.122	0.01	Nucleus	Other
CALD1	Caldesmon 1	−2.125	6.57E-06	Cytoplasm	Other
JPT1	Jupiter microtubule-associated homolog 1	−2.125	1.65E-05	Nucleus	Other
MCMDC2	Minichromosome maintenance domain–containing 2	−2.126	0.000272	Other	Other
TCEANC2	Transcription elongation factor A N-terminal and central domain–containing 2	−2.127	0.000308	Other	Other
GPAM	Glycerol-3-phosphate acyltransferase, mitochondrial	−2.133	0.00555	Cytoplasm	Enzyme
SPSB3	Spla/ryanodine receptor domain and SOCS box–containing 3	-2.141	0.00241	Cytoplasm	Other
CCIN	Calicin	−2.186	0.00688	Cytoplasm	Other
PHLPP1	PH domain and leucine rich repeat protein phosphatase 1	−2.186	0.00688	Cytoplasm	Enzyme
TCERG1	Transcription elongation regulator 1	−2.186	0.00688	Nucleus	Transcription regulator
TUBB	Tubulin beta class I	−2.186	0.000519	Cytoplasm	Other
TUBB4A	Tubulin beta 4A class iva	−2.186	0.000519	Cytoplasm	Other
TUBB4B	Tubulin beta 4B class ivb	−2.186	0.000519	Cytoplasm	Other
CDCA7L	Cell division cycle–associated 7 like	-2.207	2.73E-05	Nucleus	Other
EXOC7	Exocyst complex component 7	−2.207	1.11E-16	Cytoplasm	Transporter
USP13	Ubiquitin-specific peptidase 13	−2.207	0.00139	Cytoplasm	Peptidase
DCPS	Decapping enzyme, scavenger	−2.221	0.000555	Nucleus	Enzyme
HSP90AB1	Heat shock protein 90 alpha family class B member 1	−2.221	4.93E-06	Cytoplasm	Enzyme
HSP90AB2P	Heat shock protein 90 alpha family class B member 2, pseudogene	−2.221	4.93E-06	Cytoplasm	Other
PRKY	Protein kinase Y-linked (pseudogene)	−2.221	0.000555	Other	Other
PFN2	Profilin 2	−2.242	0.00139	Cytoplasm	Enzyme
RFC4	Replication factor C subunit 4	−2.242	0.00139	Nucleus	Other
CASP8AP2	Caspase 8–associated protein 2	−2.309	1.11E-08	Nucleus	Transcription regulator
ITSN1	Intersectin 1	−2.309	0.00134	Cytoplasm	Other
WDR59	WD repeat domain 59	−2.309	2.55E-05	Cytoplasm	Transporter
TTN	Titin	−2.321	0.000182	Cytoplasm	Kinase
AMPD3	Adenosine monophosphate deaminase 3	−2.404	0.00298	Cytoplasm	Enzyme
TBC1D32	TBC1 domain family member 32	−2.404	0.0368	Other	Other
VCAN	Versican	−2.404	0.000269	Extracellular space	Other
RNF213	Ring finger protein 213	−2.409	0.000493	Cytoplasm	Enzyme
FUT10	Fucosyltransferase 10	−2.445	0.00856	Cytoplasm	Enzyme
DNAH7	Dynein axonemal heavy chain 7	−2.561	0.0199	Cytoplasm	Other
MED23	Mediator complex subunit 23	−2.561	0.00349	Nucleus	Transcription regulator
MYEF2	Myelin expression factor 2	−2.561	0.00349	Nucleus	Transcription regulator
AHNAK	AHNAK nucleoprotein	−2.599	0.00006	Nucleus	Other
CERS1	Ceramide synthase 1	−2.599	7.36E-08	Cytoplasm	Enzyme
DLGAP4	DLG-associated protein 4	−2.599	0.00006	Plasma membrane	Other
DMRT2	Doublesex and mab-3–related transcription factor 2	−2.599	0.00006	Nucleus	Transcription regulator
ERG	ETS transcription factor ERG	−2.599	0.00006	Nucleus	Transcription regulator
F8	Coagulation factor VIII	−2.599	0.00006	Extracellular space	Other
FBL	Fibrillarin	−2.599	0.00006	Nucleus	Enzyme
FKBP8	FKBP prolyl isomerase 8	−2.599	0.00006	Cytoplasm	Other
IP6K2	Inositol hexakisphosphate kinase 2	−2.599	7.36E-08	Cytoplasm	Kinase
MGLL	Monoglyceride lipase	−2.599	7.36E-08	Plasma membrane	Enzyme
MMP2	Matrix metallopeptidase 2	−2.599	0.00006	Extracellular space	Peptidase
NAXE	NAD(P)HX epimerase	−2.599	0.00006	Extracellular space	Enzyme
OGA	O-glcnacase	−2.599	0.00006	Cytoplasm	Enzyme
PPP1R42	Protein phosphatase 1 regulatory subunit 42	−2.599	0.00006	Cytoplasm	Other
PYGM	Glycogen phosphorylase, muscle associated	−2.599	0.00006	Cytoplasm	Enzyme
RARS2	Arginyl-trna synthetase 2, mitochondrial	−2.599	7.36E-08	Cytoplasm	Enzyme
RGS12	Regulator of G protein signaling 12	−2.599	7.36E-08	Nucleus	Enzyme
RGSL1	Regulator of G protein signaling–like 1	−2.599	9.83E-11	Other	Other
SOGA3	SOGA family member 3	−2.599	7.36E-08	Other	Other
TAX1BP1	Tax1-binding protein 1	−2.599	0.00006	Cytoplasm	Other
TGOLN2	Trans-golgi network protein 2	−2.599	0.00006	Cytoplasm	Other
TP53	Tumor protein p53	−2.599	7.36E-08	Nucleus	Transcription regulator
HSP90AA1	Heat shock protein 90 alpha family class A member 1	−2.64	1.33E-06	Cytoplasm	Enzyme
YKT6	YKT6 v-SNARE homolog	−2.704	0.00022	Cytoplasm	Enzyme
FER1L5	Fer-1–like family member 5	−2.795	0.0316	Other	Other
DST	Dystonin	−2.969	0.0102	Plasma membrane	Other
SCNN1A	Sodium channel epithelial 1 alpha subunit	−2.969	0.0366	Plasma membrane	Ion channel
GLYATL3	Glycine-N-acyltransferase–like 3	−12.023	0.00137	Other	Other
TGFB2	Transforming growth factor beta 2	−12.023	0.00137	Extracellular space	Growth factor
NPM1	Nucleophosmin 1	−20.153	1.37E-09	Nucleus	Transcription regulator

Proteins within the PPARα/RXRα canonical pathway included HLA-B/C, LSM14A, ASPM whose phosphorylation was increased (Figs. 3*C*, S2, *A*–*C*, Tables 1 and S10). This pathway also included NPM1, p53, HSP90AA1, and HSP90AB1 whose phosphorylation was decreased (Figs. 3*C*, S2, *D*–*F*, Tables 1 and S10). PKR-regulated pathway included HSP90AA1, HSP90AB1, MYD88, NPM1, STAT3, and TP53 (supplemental Table S10). NPM1 was dephosphorylated on Ser-125 residue which resides within ^104^*c*GSGPVHISGQHLVAVEEDAE*s*EDEEEEDVK^134^ peptide (Fig. 3, *D* and *E*).

**Fig. S3:**
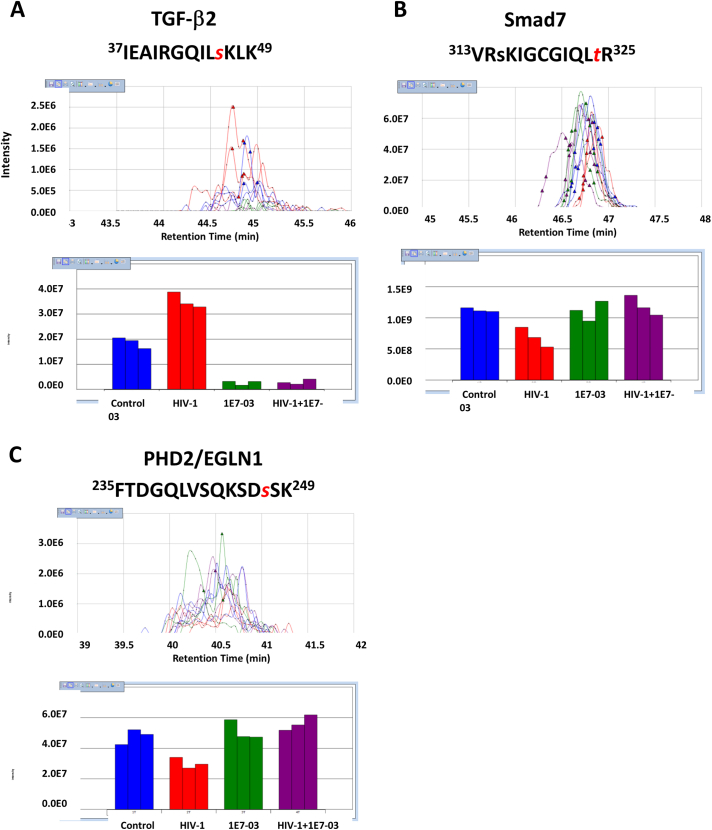
**Label-free quantification of representative phosphoproteins in TGF-b signaling.** SIEVE quantitative data of phosphopeptides ^37^IEAIRGQIL*s*KLK^49^from TGF-b2 (panel *A*), ^313^VRsKIGCGIQL*t*R^325^ from Smad7 (panel *B*) and ^235^FTDGQLVSQKSD*s*SK^249^from PHD2/EGLN1 (panel *C*). Ion elution profiles (*top*) and integrated intensities (*bottom*) are shown in blue for non-infected cells, in red for HIV-1 infected cells, in green for non-infected cells plus 1E7-03 treatment and in purple for HIV-1 infected cells plus 1E7-03 treatment. The data was shown in triplicate.

Proteins within TGF-β pathway affected by 1E7-03 (Fig. 3, *B* and *F*) included TGF-β2 (supplemental Fig. S3*A*), SMAD7 (supplemental Fig. S3*B*), and PD2 (supplemental Fig. S3*C*). Telomerase signaling pathway includes HSP90AA1, HSP90AB1, MAP2K2, and TP53 proteins (supplemental Table S10).

**Fig. S4:**
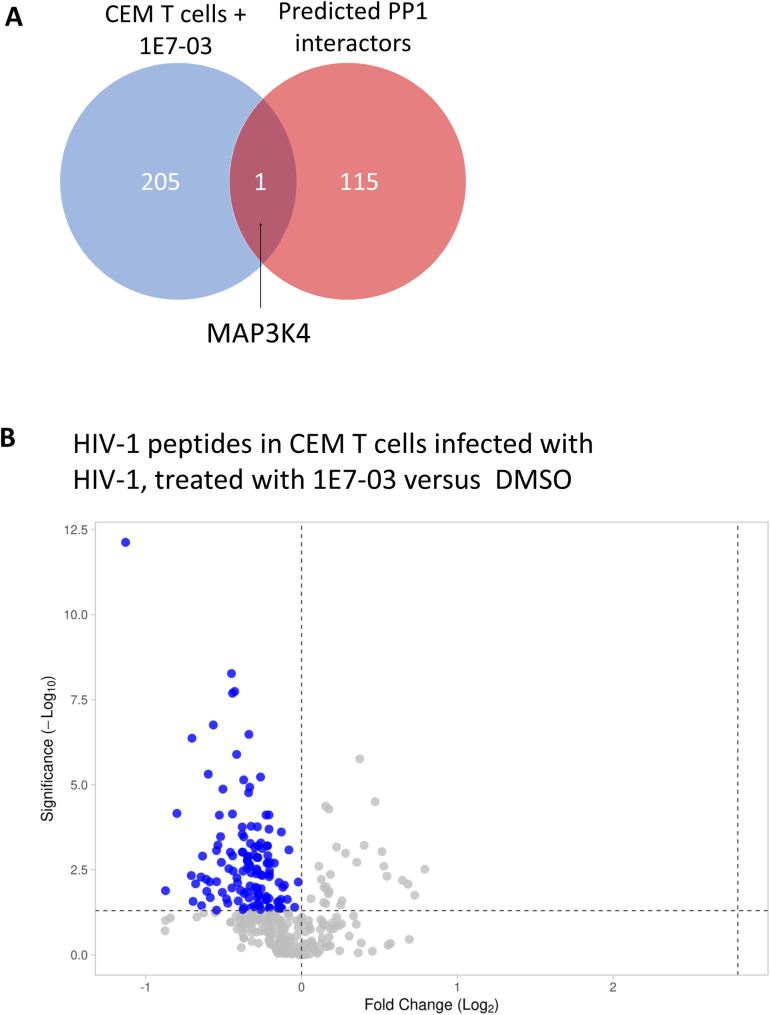
*A,*Venn diagram showing comparison of phosphorylated proteins from CEM T cells treated with 1E7-03 and predicted PP1 interacting proteins.**)** Volcano plot shows HIV-1 peptides quantified by SIEVE 2.1 in HIV-1 infected CEM T cells comparing to the infected cells treated with 1E7-03. Blue color indicated downregulation. Venn diagram showing comparison of phosphorylated proteins from CEM T cells treated with 1E7-03 and predicted PP1 interacting proteins. *B,*Volcano plot shows HIV-1 peptides quantified by SIEVE 2.1 in HIV-1 infected CEM T cells comparing to the infected cells treated with 1E7-03. Blue color indicated downregulation.

We compared proteins upphosphorylated and downphosphorylated in CEM T cells infected with HIV-1 and treated with 1E7-03 with the previously set of PP1-interacting proteins (28) that were identified in a broad *in silico* screening. Only one protein, MAP kinase kinase kinase 4 (MAP3K4), was found to overlap between the predicted PP1 interactors and the phosphoproteins in 1E7-03–treated CEM T cells (supplemental Fig. S4*A*). We also assessed levels of HIV-1 peptides in the infected CEM cells treated with 1E7-03 which showed overall reduction (supplemental Fig. S4*B*).

Taken together, modulation of protein phosphorylation in multiple pathways including PPARα/RXRα and TGF-β pathways is likely to contribute to HIV-1 inhibition by 1E7-03.

### 1E7-03 Does Not Affect the Overall Host Proteins Expression Level

**Fig. 4:**
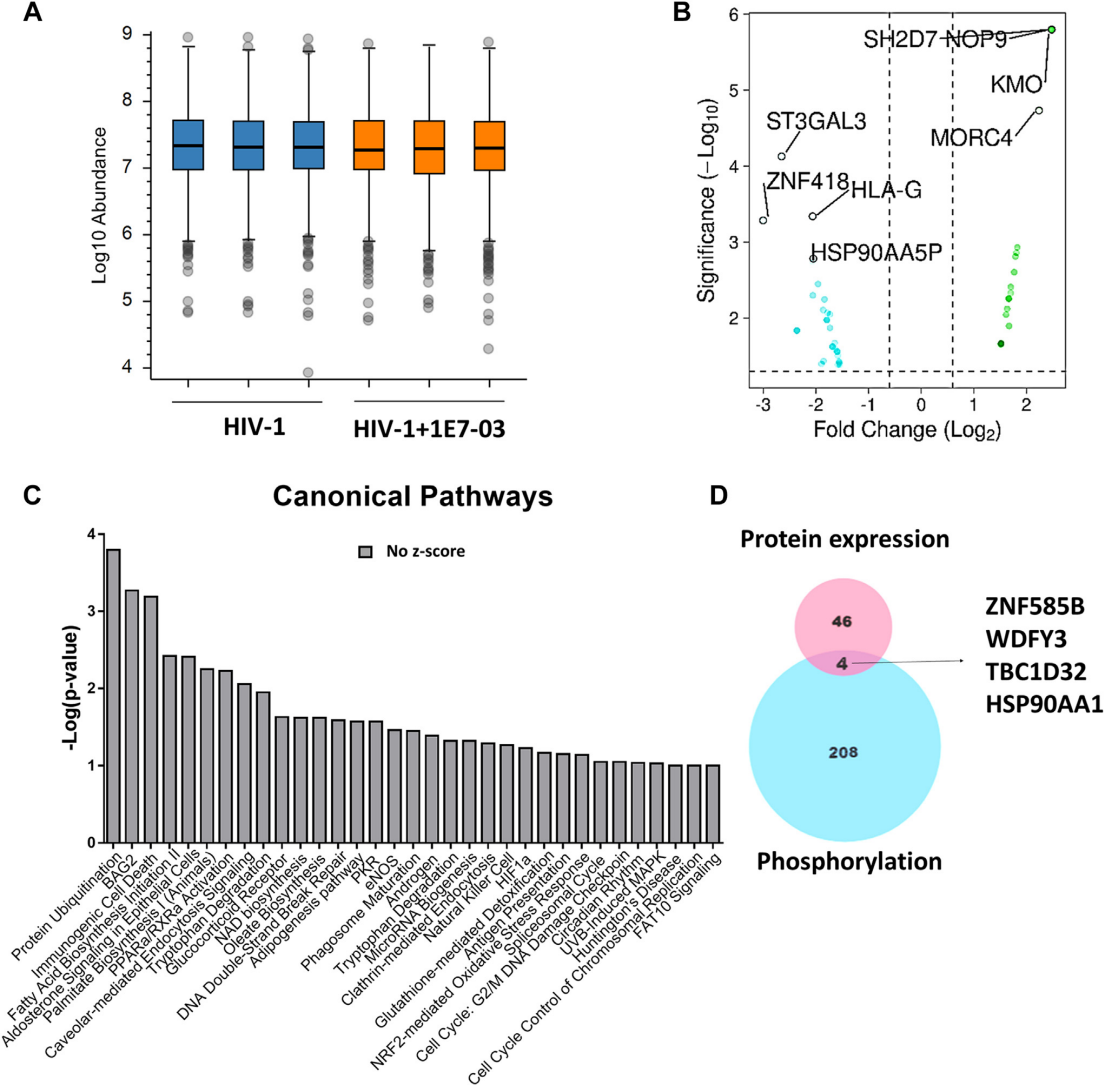
**1E7-03 has no effect on host proteins expression.***A*, the overall protein abundance in HIV-1–infected cells with or without 1E7-03 treatment. *Blue* color represents DMSO control group, and *orange* color represents 1E7-03–treated group. *B*, volcano map of host protein expression whose expression was changed by 1E7-03. *Cyan* and *green* dots represent host proteins significantly downregulated or upregulated by 1E7-03 with cutoff >1.5 fold and *p* < 0.05. *C*, canonical pathways analysis of host protein expression affected by 1E7-03. Color (*gray*) indicate that pathways had no activity. *D*, Venn diagram showing comparison of changes in expression induced by 1E7-03. HIV-1, human immunodeficiency virus-1; DMSO, dimethyl sulfoxide.

We next analyzed the effect of 1E7-03 on host protein expression levels by conducting label-free quantitative proteome analysis in HIV-1–infected CEM T cells with or without 1E7-03 treatment. No changes in overall protein expression levels were observed (Fig. 4*A*). Volcano map analysis of total 5311 detected proteins (1.5-fold cutoff and *p*-value < 0.05) showed 21 upregulated proteins (green dots, Figs. 4*B*) and 29 downregulated proteins (cyan dots, Fig. 4*B* and supplemental Table S11). This observation indicated that expression of most host proteins was not significantly affected by 1E7-03 treatment. These differentially expressed proteins were further analyzed by IPA which showed that no major regulatory pathway was affected (Fig. 4*C* and Supplemental Table S12). Analysis of individual proteins also did not show any differentially expressed proteins related to HIV-1 replication regulation except heat shock proteins (1.56-2.049-fold reduction) and HLA-G (2.062-fold reduction) (supplemental Table S11). Comparison of the 50 differentially expressed proteins (supplemental Table S11) with the 212 proteins that had changes in their phosphorylation (Table 1) pointed to four overlapping proteins, including Hsp90AA1 (Fig. 4*D*). Thus, phosphorylation of Hsp90AA1 may be affected indirectly through the changes in its expression level. Taken together, 1E7-03 is likely to achieve antiviral effect by inducing protein phosphorylation and dephosphorylation rather than affecting the expression level of host proteins.

### Phosphorylation of NPM1 Ser-125 Residue Contributes to HIV-1 Transcription Activation

**Fig. 5:**
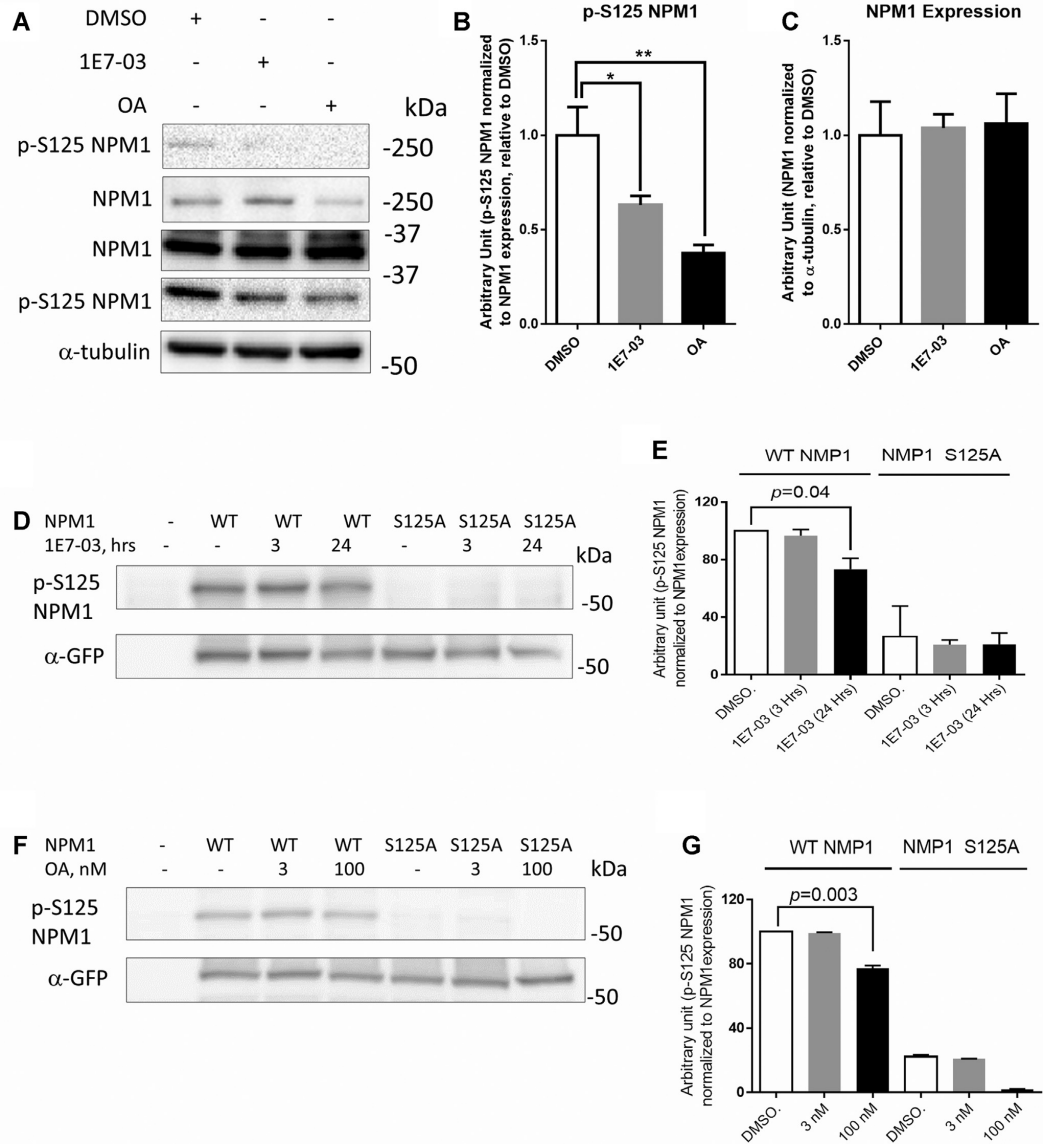
**Effect ofPP1 inhibitors onNPM1 Ser-125 phosphorylation.***A*, 1E7-03 downregulates NPM1 Ser-125 phosphorylation. CEM T cells were treated with 10 μM 1E7-03 for 24 h and 100 nM okadaic acid (OA) for 2 h as a positive control. The protein expression and phosphorylation of NPM1 Ser-125 was analyzed by Western blotting with antibodies against NPM1 and Ser-125 phospho-specific antibodies (p-S125 NPM1). *B* and *C*, quantification of Ser-125–phosphorylated NPM1 and NPM1 performed using Prism 6 from three independent experiments. *Asterisks* indicate *p* < 0.05 (∗) and *p* < 0.01 (∗∗). *D*, effect of short incubation with 1E7-03 on NMP1 phosphorylation. HEK293T cells were transfected with vectors expressing GFP-tagged WT NPM1 and NPM1 S125A mutant and treated with 10 μM 1E7-03 for 3 h (short treatment) and 24 h (long treatment). The phosphorylation of NPM1 Ser-125 and protein expression was analyzed by Western blotting with antibodies for p-S125 NPM1 and GFP. *E*, quantification of results from panel *D* by Prism 6 from three independent experiments. *F*, effect of low concentration of OA on NPM1 phosphorylation. HEK293T cells were transfected as in panel *D* and treated with 3 nM or 100 nM OA for 2 h. Phosphorylation of NPM1 Ser-125 and protein expression were analyzed with antibodies for p-S125 NPM1 and GFP. *G*, quantification of the data form panel F was performed using Prism 6 from three independent experiments. HIV-1, human immunodeficiency virus-1; NPM1, nucleophosmin.

We next validated NPM1 phosphorylation and assessed its biological effect. We chose NPM1 as its dephosphorylation was the strongest among the phosphorylation changes that were induced by 1E7-03 (Fig. 3*A*). To confirm that PP1 was involved in NPM1 phosphorylation, we analyzed endogenous NPM1 phosphorylation in CEM T cells treated with 10 μM 1E7-03 (24 h) or 100 nM OA (2 h), which served as positive control as OA inhibits PP1 at higher 10 nM concentrations and PP2A at lower 3 nM concentration (29). NPM1 expression levels and its Ser-125 phosphorylation were analyzed by immunoblotting using antibodies against NPM1 and NPM1 phosphorylated on Ser-125 (p-S125 NPM1). Compared to DMSO control, both 1E7-03 and OA treatments significantly reduced NPM1 phosphorylation on Ser-125 residue without affecting its protein expression level (∗*p* < 0.015 for 1E7-03 and ∗∗*p* < 0.002, Fig. 5, *A*–*C*). We also tested the effect of short-term 3 h treatment with 1E7-03 comparing to 24 h treatment. HEK293T cells were transfected with vectors expressing NPM1-GFP or NPM1 S125A-GFP. While 24 h treatments led to decreased NPM1 phosphorylation, there were no changes in NPM1 phosphorylation at 3 h treatment (Fig. 5, *D* and *E*), We also analyzed the effect of lower concentration of OA, inhibitory for PP2A, on NPM1 phosphorylation using NPM1-GFP expressed in HEK293T cells. Comparing to the reduced NPM1 phosphorylation at 100 nM OA concentration, there was no effect at 3 nM OA treatments (Fig. 5, *F* and *G*).

**Fig. 6:**
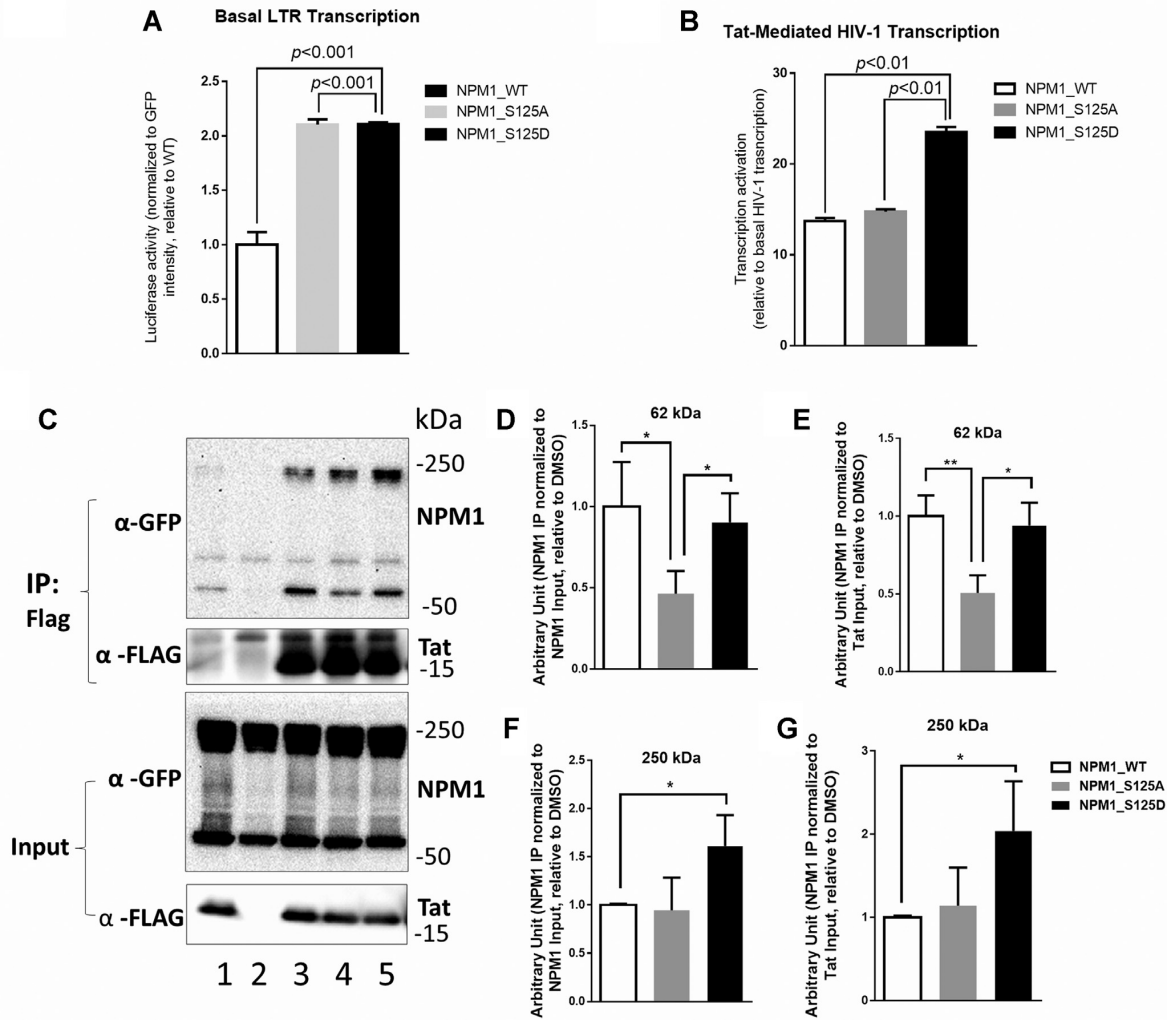
**Effect of NPM1 Ser-125 phosphorylation on HIV-1 transcription.***A* and *B*, effect of expression of NPM1 and its phosphorylation mutants (S125A and S125D) on basal (panel *A*) and Tat-mediated HIV-1 transcription (panel *B*). HEK293T cells were transfected with vectors expressing the indicated GFP-tagged NPM1 mutants and cotransfected with vectors expressing HIV-1 LTR-luciferase reporter (panel *A*) or HIV-1 LTR-luciferase reporter and FLAG-tagged Tat expression vector (panel *B*). Luciferase activity was analyzed using Luclite plus Reporter Gene Assay and normalized to GFP intensity. All data were shown in triplicates as transcription activation fold related to the WT NPM1. *C*, effect of NPM1 Ser-125 phosphorylation on the interaction with Tat protein. GFP-tagged NPM1 (WT, S125A, and S125D) and Flag-tagged HIV-1 Tat were expressed in 293T cells. Tat was precipitated with anti-Flag antibody, resolved on SDS-PAGE, and immunoblotted with anti-GFP to detect NPM1 and anti-Flag antibodies to detect Tat. Lane 1, IgG control; Lane 2, No Tat; Lane 3, Tat+NPM1 WT; Lane 4, Tat+NPM1 S125A; Lane 5, Tat+NPM1 S125D. *D*–*G*, quantification of the coimmunoprecipitation data from three independent experiments. NPM1’s 62 kDa and 250 kDa isoforms were normalized to NPM1 and Tat input, respectively. Asterisks indicate *p* < 0.05 (∗) and *p* < 0.01 (∗∗). HIV-1, human immunodeficiency virus-1; LTR, long terminal repeat; NPM1, nucleophosmin.

**Fig. S5:**
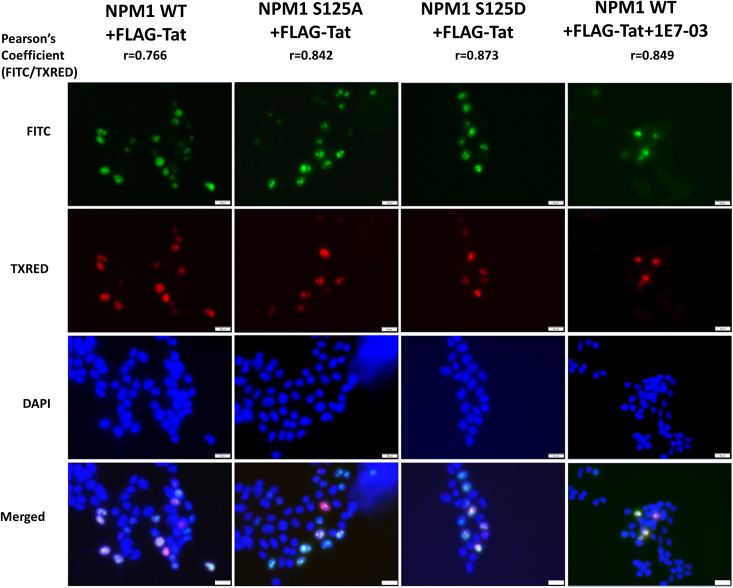
**The phosphorylation of NPM1 at Ser-125 does not affect nuclear colocalization of NPM1 and Tat.** 293 cells were co-transfected with GFP-tagged NPM1 WT, S125A or S125D vector along with Flag-tagged Tat vector following the manufacturer’s protocol of Lipofectamine 3000 (Invitrogen). Additional transfected group of NPM1 WT and FLAG-Tat was treated with 1E7-03. At 24 hrs post-transfection, the cells were fixed with 4% paraformaldehyde (PFA), permeabilized with 0.25% Triton X-100, immunoblotted with antibodies against GFP (ab290, Abcam) and FLAG (F1804, Sigma-Aldrich), and incubated with corresponding secondary antibodies conjugated with Alex Fluor 488 and 594 dyes (Thermo Fisher Scientific). Cells were also stained with Hoechst (Thermo Fisher Scientific). Stained cells were photographed on Olympus IX73 using filter for FITC, TXRED and DAPI fluorescence at 600X magnification. Person’s correlation analysis was conducted in ImageJ.

**Fig. S6:**
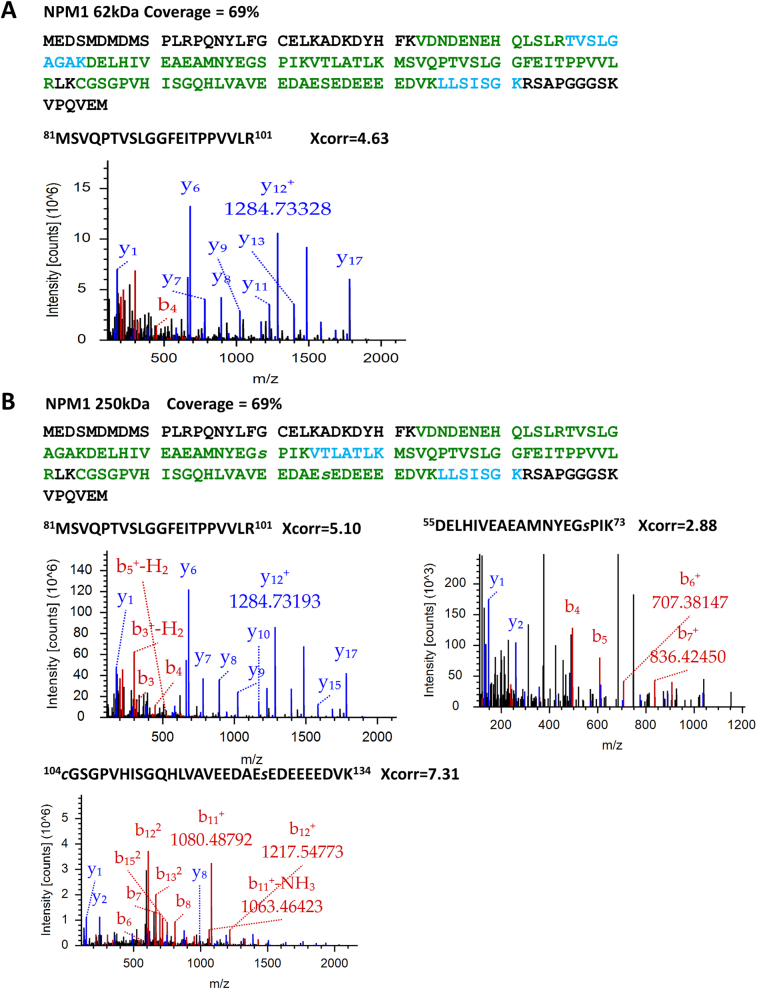
**LC-FT/MS analysis of NPM1 phosphorylation.***A*, Peptide mapping of NPM1 from 62 kDa band with 69% coverage. Peptides identified with high and median probability are shown in green and blue, respectively. Representative MS/MS spectrum of peptide ^81^MSVQPTVSLGGFEITPPVVLR^101^ derived from NPM1. The colored peaks indicate matched MS/MS fragments. Blue and red colors indicate y and b ions, respectively. *B*, Peptide mapping of NPM1 from 250kDa band with the same coverage. Besides peptide ^81^MSVQPTVSLGGFEITPPVVLR^101^, representative MS/MS spectra of two phosphopeptides ^55^DELHIVEAEAMNYEG*s*PIK^73^ and ^104^*c*GSGPVHISGQHLVAVEEDAE*s*EDEEEEDVK^134^ are shown.

**Fig. S7:**
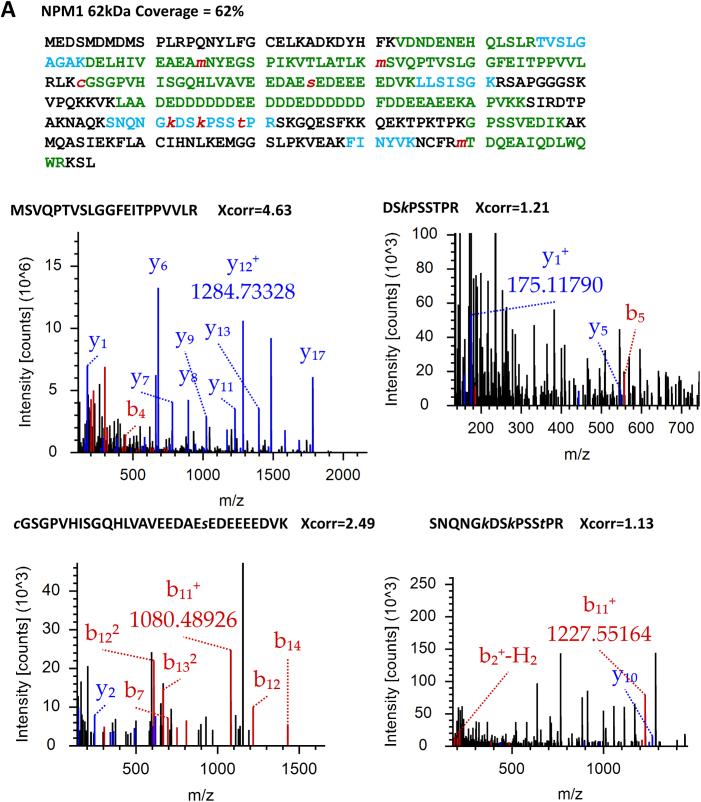


Previously, NPM1 was shown to facilitate nuclear localization of HIV-1 Tat, thus enhancing viral transcription (30). HIV-1 transcription activation was evaluated by coexpressing GFP-tagged NPM1 WT, NPM1 S125A mutant, or NPM1 S125D mutant along with HIV-1 long terminal repeat–luciferase reporter without or with Tat-expressing vector in HEK293T cells. NPM1 phosphorylation did not affect its nuclear colocalization with Tat as NPM1, NPM1 S125A, and NPM1 S125D mutants showed similar nuclear colocalization with Flag-Tat, when quantified with Pearson’s correlation analysis (supplemental Fig. S5). Coexpression of NPM1 S125A or NPM1 S125D mutants increased HIV-1 basal transcription (2.1-fold, Fig. 6*A*). Coexpression of NPM1 S125D mutant induced Tat-mediated HIV-1 transcription (1.77-fold increase, Fig. 6*B*) compared to the expression of WT NPM1. In contrast, expression of nonphosphorylation–mimicking NPM1 S125A mutant had no effect on Tat-activated HIV-1 transcription (Fig. 6*B*). As NPM1 was shown to interact with Tat, we tested their binding by coimmunoprecipitation of GFP-tagged NPM1 (WT, S125A, and S125D) and Flag-tagged HIV-1 Tat. NPM1 was migrating as 62-kDa monomeric form and a larger 250-kDa form that might represent an oligomeric form of NPM1 or its covalently modified species (Fig. 6*C*). Both protein bands were excised and further analyzed by nano LC-MS/MS that confirmed the presence of NPM1 with 69% coverage (supplemental Fig. S6, *A* and *B*). Phosphorylation of Ser-70 and Ser-125 residues was detected in 250-kDa species (supplemental Fig. S6*B*). Analysis of NPM1 acetylation showed that 62-kDa species was both acetylated and phosphorylated, whereas the 250-kDa species was phosphorylated but not acetylated (supplemental Fig. S7). Analysis of Tat interaction with NPM1 showed that 62-kDa form interaction was disrupted when nonphosphorylated NPM1 S125A mutant was used, whereas the phosphorylation mimicking NPM1 S125D mutant bound similar to the WT NPM1 (Fig. 6, *D*, and *E*). In contrast, the 250-kDa species of NPM1 showed equal interaction of WT and S125A mutant with Tat but enhanced interaction of NPM1 125D mutant (Fig. 6, *F* and *G*). These observations suggest that dephosphorylated and acetylated monomeric form of NPM1 might not bind Tat efficiently unless NPM1 is phosphorylated. In the oligomeric nonacetylated form, NPM1 phosphorylation enhances Tat binding. Taken together, these results show that downregulation of NPM1 Ser-125 phosphorylation by 1E7-03 is likely to contribute to HIV-1 transcription inhibition.

### Aurora Kinases Inhibition as a Drug Target for HIV-1 Replication

NPM1 is phosphorylated on Ser-125 by several kinases including Aurora A, Aurora B, and casein kinase 2 (CK2) (31). To further confirm the role of Aurora kinases in HIV-1 replication, Aurora A inhibitor I and Aurora B inhibitor, Barasertib, were tested in one round HIV-1 infection and on cellular cytotoxicity using 1E7-03 as a reference. Aurora A inhibitor I and Barasertib suppressed one round HIV-1 infection (IC_50_ = 7.8 μM and IC_50_= 13.7 μM, respectively) (Fig. 7*A*). The effects of Aurora kinase inhibitors were less pronounced comparing to the effect of 1E7-03 (IC_50_ = 5.3 μM) (Fig. 7*A*). Cell viability analysis showed minimal toxicity for 1E7-03 and Barasertib in CEM T cells (CC_50s_>100 μM, Fig. 7*B*), whereas Aurora A inhibitor I showed significant toxicity (CC_50_ = 10 μM, Fig. 7*B*).

**Fig. 7:**
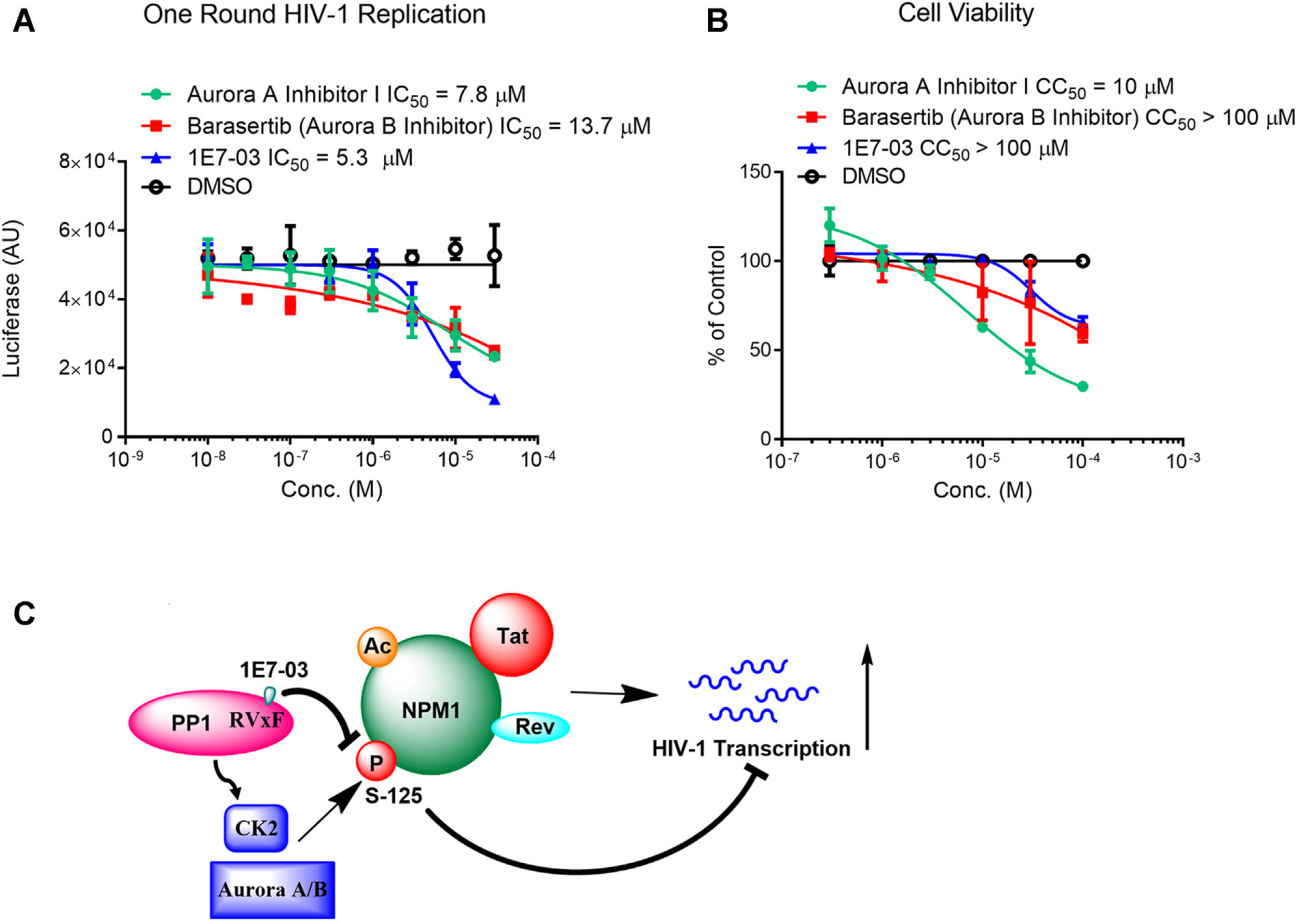
**Inhibition of upstream NPM1 kinases blocks HIV-1 infection.***A*, inhibition of one round HIV-1 infection by Aurora A inhibitor and Aurora B inhibitors and 1E7-03 (control). CEM T cells were infected with HIV-1-LUC-G and treated with the indicated inhibitor concentrations for 24 h. HIV-1–expressed luciferase activity is shown as mean ± SD of triplicates. *B*, cytotoxicity of Aurora A and Aurora B inhibitors and 1E7-03 (control). CEM T cells were treated with the indicated inhibitor concentrations for 24 h. The cell viability was analyzed by MTT assay and plotted as percent relative to DMSO-treated control. Data are the mean ± SD of triplicates. *C*, proposed molecular mechanism of NPM1 in HIV-1 transactivation. During HIV-1 infection, acetylation and phosphorylation of NPM1 enhance the interaction with Tat-facilitating Tat-mediated HIV-1 transcription activation. Phosphorylation of NPM1 at Ser-125 is mediated by Aurora A/B or CK2 kinases which might be indirectly affected by PP1. HIV-1, human immunodeficiency virus-1; NPM1, nucleophosmin; PP1, protein phosphatase 1; DMSO, dimethyl sulfoxide; CK2, casein kinase 2.

Taken together, this study shows that HIV-1 inhibition by 1E7-03 might be the outcome of complex attenuation of host protein phosphorylation within the PPARα/RXRα and TGF-β pathways that involves dephosphorylation of NPM1.

## Discussion

HIV-1 replication takes advantage of posttranslational modifications of many cellular proteins, including NPM1 (schematics depicted in Fig. 7*C*). NPM1 Ser-125 phosphorylation is likely to be regulated by various kinases and phosphatases including PP1, Aurora A/B, and CK2. As we showed here, PP1-targeting 1E7-03 blocks HIV-1 replication in part by downregulating NPM1 phosphorylation that may impede NPM1–Tat interaction (Fig. 7*C*). Decreased NMP1 phosphorylation in the cells treated with 1E7-03 or high concentration of OA indicate that PP1 is involved in the control of NPM1 phosphorylation. However, the effect of PP1 on NPM1 is indirect as NMP1 phosphorylation decreases upon PP1 inhibition. The NPM1 phosphorylation is mediated by one of the NPM1 kinases, and PP1 is likely affecting an upstream kinase. As NPM1 phosphorylation contributes to the inhibition of HIV-1 transcription, targeting NPM1 phosphorylation, indirectly with 1E7-03 or directly with a kinase inhibitor, such as Barasertib, can serve as a future avenue for HIV-1 transcription inhibition.

PP1 has traditionally been overlooked as a drug target because of the conserved catalytic site of PP1 that makes it nearly impossible for achieving selective enzymatic inhibition without having global cellular effect (32). The existing enzymatic PP1 inhibitors, such as microcystin, nodularin, OA, and tautomycin, are toxic to the host cells. However, as the substrate specificity of PP1 is mediated by its regulatory subunits, there is an opportunity for developing noncatalytic PP1 inhibitors that might specifically target individual PP1 holoenzymes (17, 18). In this study, we conducted for the first time a comprehensive analysis of host protein phosphorylation in cells treated with PP1-targeting 1E7-03 molecule using quantitative mass spectrometry. Different enrichment strategies were employed including Fe-NTA and TiO_2_ enrichment and compared to the nonenriched peptide samples. While only 14% of the cells were infected with HIV-1, 1E7-03 seems to have more robust effect in HIV-1–infected cells, implying intricate interactions among virus, host, and the drug.

The expected effect of 1E7-03 is the increased protein phosphorylation as the compound affects PP1-mediated dephosphorylation and may enhance phosphorylation of PP1-targeted proteins. Label-free quantitative phosphoproteome analysis showed that 1E7-03 induced phosphorylation of several proteins from several pathways including PPARα/RXRα and TGF-β pathways. Also, unexpectedly, we observed decreased phosphorylation of several proteins that also were part of the same pathways. Both pathways have been implicated in the regulation of HIV-1 replication. In the PPARα/RXRα canonical pathway, proteins with increased phosphorylation included HLA-B/C, LSM14A, and ASPM. In the PPARα/RXRα pathway, proteins with decreased phosphorylation included RGSL1, p53 (coded by *TP53*), HSP90AA1, TGF-β2, and NPM1. HLA-B∗27, HLA-B∗57, and HLA-B∗58 are well-known protective alleles that contribute to the suppression of HIV-1 replication and prevent progression toward AIDS (33). LSM14A initiates cellular antiviral response in the early phase of viral infection by regulating MITA level in a cell-specific manner (34, 35). ASPM is a binding partner of PP1 and contains RVxF motif (KVSF) which is phosphorylated by Aurora B kinase during mitosis (36). TGF-β superfamily of cytokines include three isoforms (β1, β2, and β3) that elicit similar biological responses through the same receptor (37). HIV-1–infected patients exhibit higher levels of TGF-β1 which is considered a major driver of immunosuppression (38). TGF-β1 might also induce apoptosis of CD4+ T lymphocytes following macrophage-tropic (R5) HIV-1 infection, by reducing levels of anti-apoptotic factors as well as by increasing apoptosis-inducing factors (39). HIV-1 Tat protein induces mRNA expression and secretion of TGF-β1, which is also induced by HIV-1 glycoprotein (gp) 160 (38). TGF-β2 has been shown to exert suppressive effects on IL-2–dependent T-cell growth (40). TGF-β isoforms can exert their effects through either SMAD-dependent or SMAD-independent signaling pathways (41).

We validated the role of NPM1 phosphorylation in HIV-1 transcription as this was the most significantly affected protein whose phosphorylation decreased in 1E7-03–treated cells. NPM1 has been reported to play a role in nuclear localization of HIV-1 Tat and Rev proteins and its acetylation was shown to be required for HIV-1 transcription activation (30, 42). NPM1 Ser-125 residue is phosphorylated by cell cycle–related Ser/Thr protein kinases such as Aurora A, Aurora B, and CK2 (43). NPM1 is acetylated by p300 (30, 42). To the best of our knowledge, NPM1 phosphorylation on Ser-125 has not been linked to HIV-1 transcription regulation nor connected to PP1. NPM1 phosphorylation having effect on the interaction with HIV-1 Tat has not yet been described either. Our results indicate that monomeric form of NPM1 might be acetylated but Tat binding of this form is not affected by phosphorylation. In contrast, oligomeric form of NPM1 was not acetylated and its phosphorylation enhanced Tat binding. It is possible that acetylation and phosphorylation are two independent mechanisms that both may contribute to HIV-1 transcription activation. Further analysis is needed to analyze crosstalk of phosphorylation and acetylation and whether both modifications contribute independently to HIV-1 activation.

Previously, p53 was shown to suppress HIV-1 replication through the activation of PKR and phosphorylation of HIV-1 Tat protein on Ser-46 residue (12). PKR regulatory pathway was identified in our study as a pathway targeted by 1E7-03. P53 was previously shown to reactivate HIV-1 replication in latently infected U1 cells by modulating PI3K/Akt and MAPK Erk/p38 signaling pathways (44, 45). P53 also interacts with HIV-1 Vif protein which noncanonical function includes G2 cell cycle arrest (46). Phosphorylation and dephosphorylation of p53 within N- and C-terminal domains leads to its stabilization and activation (47). PP1 dephosphorylates p53 Ser-15 residue and this dephosphorylation is blocked by UV light which facilitates PP1 binding to GADD34 (48). The p53 regulated gene, p21, is expressed in HIV-1 elite controllers (49). The p21 is also linked to the phosphorylation of the SAM domain and HD domain–containing protein 1 (SAMHD1) (50). Induction of p21 expression and downregulation of CDK2 can also affect HIV-1 reverse transcription (RT) as CDK2 phosphorylates HIV-1 RT (51). Additionally, p21 controls the expression of ribonucleotide reductase 2 that may lower dNTP pool and impair HIV-1 RT (52, 53, 54). Induction of p21 expression by iron chelators can contribute to the inhibition of HIV-1 transcription (55). p21 also interferes with the early stage of HIV-1 replication in primary microglia and astrocytes (56).

Hsp90 activates HIV-1 transcription by inducing activities of several host transcription factors including NF-κB, NFAT, and STAT5 (57). Hsp90 activity is regulated by Hsp90α phosphorylation on Thr-36, Thr-90, Ser-231, and Ser-263 and Hsp90β phosphorylation on Ser-365 (58, 59). Hsp90 is phosphorylated by CK2, PKA and dephosphorylated by PP5, PP1, and PP2A (59, 60). Taken together, modulation of protein phosphorylation in PPARα/RXRα pathway by 1E7-03 is likely to contribute to HIV-1 inhibition.

SMAD7, a potent inhibitor of TGF-β–dependent signaling, was previously shown to be phosphorylated on Ser-206 and Ser-249 residues (61, 62). The Ser-206 phosphorylation, adjacent to the PPxY motif, enhances SMAD7 binding to WW4 domain of WWP2, an E3 ubiquitin ligase (62). SMAD7 interacts with GADD34 and facilitates recruitment of PP1 catalytic subunit to dephosphorylate TGF-β type I receptor (63). TGF-β promotes formation of the ternary complex between PP1, GADD34, SMAD7, and TβRI. Hypoxia-inducible factor 1α (HIF-1α) expression is increased in HIV-1 infection (64), and TGF-β induces HIF-1α accumulation and activity by increasing HIF-1α protein stability. TGF-β specifically decreases both mRNA and protein levels of a HIF-1α–associated PHD2 (encoded by *EGLN1* gene), through the SMAD signaling pathway (65). Phosphorylation of PHD2 on Ser-125 by rapamycin (mTOR) downstream kinase P70S6K increases its activity, and dephosphorylation by protein PP2A reduces it (66). We observed increased PHD2 phosphorylation in HIV-1–infected cells, suggesting that PP1 might also be involved in the regulation of PHD2 activity in addition to PP2A. Treatment with 1E7-03 might block PHD2 Ser-246 dephosphorylation and this may lead to HIV-1 inhibition. We also observed increased phosphorylation of TAF4. As HIV-1 Tat facilitates recruitment of TBP in the absence of TAFs (67), the significance of increased TAF4 phosphorylation by 1E7-03 is yet to be clarified.

In conclusion, our study advanced the understanding of HIV-1 transcription regulatory mechanism by PP1 and pointed to PP1-targeting molecules such as 1E7-03 as viable drugs to be further developed for HIV-1 inhibition. The phosphoregulation of host proteins such as NPM1 by phosphatase or kinase-targeting small molecules may serve as new promising avenue for HIV-1 transcription inhibition.

## Data availability

The mass spectrometry raw data and Proteome Discoverer processed files are uploaded to UCSD Massive Server (https://massive.ucsd.edu/).

## Supplemental data

This article contains supplemental data.

## Conflict of interest

The authors declare no competing interests. SN is a US patent holder (US8278326B2) for inhibitors of PP1 that have been shown to slow replication of HIV-1.

## Figures and Tables

**Fig. S2.tif:**
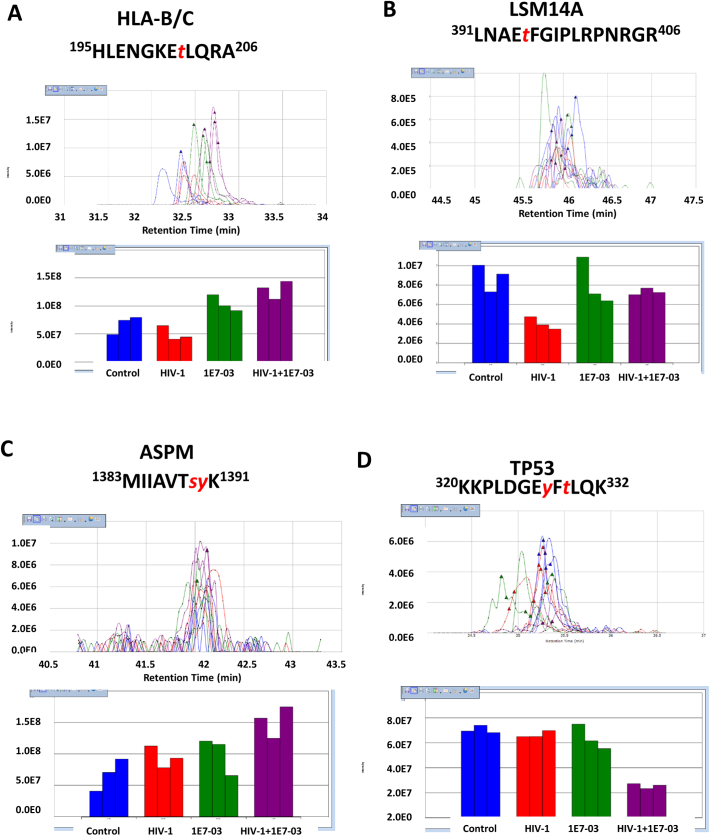


**Fig. S2 cont.tif:**
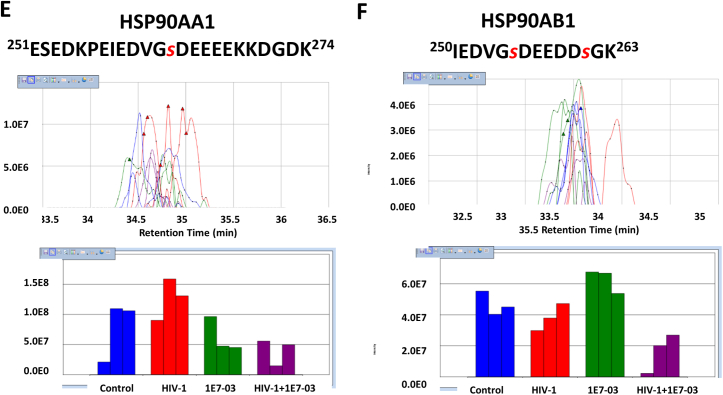
**Label-free quantification of representative phosphoproteins in PPARa/RXRa activation pathway.** SIEVE quantitative data of phosphopeptides ^195^HLENGKE*t*LQRA^206^ from HLA-B/C (panel *A*), ^391^LNAE*t*FGIPLRPNRGR^406^from LSM14A (panel *B*), ^1383^MIIAVT*sy*K^1391^from ASPM (panel *C*), ^320^KKPLDGE*y*F*t*LQK^332^ from TP53 (panel *D*), ^251^ESEDKPEIEDVG*s*DEEEEKKDGDK^274^ from HSP90AA1 (panel E) and ^250^IEDVG*s*DEEDD*s*GK^263^from HSP90AB1 (panel *F*).) Ion elution profiles (*top*) and integrated intensities (*bottom*) are shown in blue for non-infected cells, in red for HIV-1 infected cells, in green for non-infected cells plus 1E7-03 treatment and in purple for HIV-1 infected cells plus 1E7-03 treatment. The data was shown in triplicate.

**Fig. 7S cont.tif:**
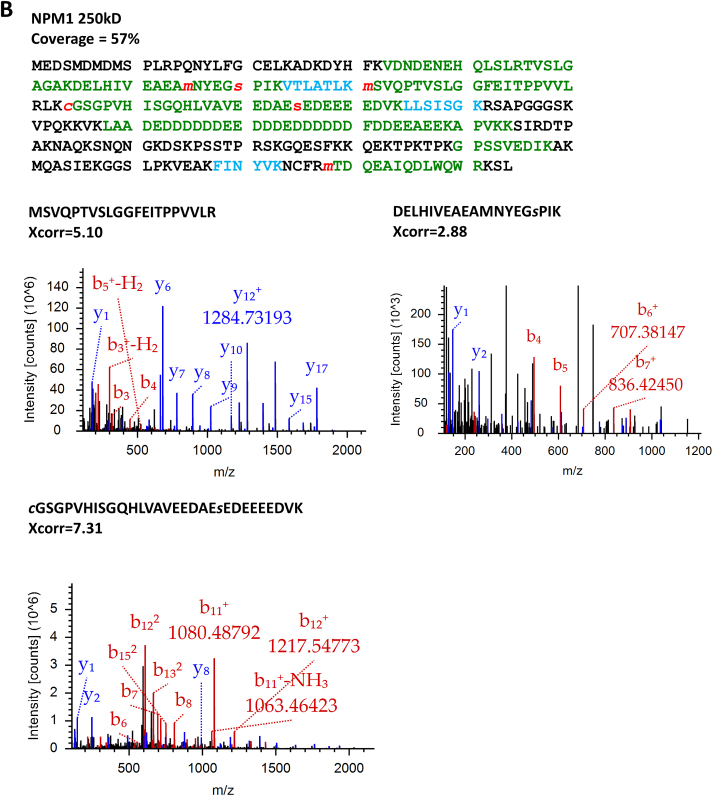
**LC-FT/MS analysis of NPM1 acetylation and phosphorylation.***A*, Peptide mapping of NPM1 from 62 kDa band with 62% coverage. Peptides identified with high and median probability are shown in green and blue, respectively. Shown representative MS/MS spectra of peptides derived from NPM1 including ^81^MSVQPTVSLGGFEITPPVVLR^101^, ^213^DSkPSSTPR^221^, ^104^*c*GSGPVHISGQHLVAVEEDAE*s*EDEEEEDVK^134^(phosphorylated) and ^207^NQNG*k*DS*k*PSS*t*PR^220^(acetylated). The colored peaks indicate matched MS/MS fragments. Blue and red colors indicate y and b ions, respectively. *B*, Peptide mapping of NPM1 from 250 kDa band with the 57% coverage. Shown are peptides ^81^MSVQPTVSLGGFEITPPVVLR^101^,^5^DELHIVEAEAMNYEG*s*PIK^73^(phosphorylated) and ^104^*c*GSGPVHISGQHLVAVEEDAE*s*EDEEEEDVK^134^ (phosphorylated).
