# Supplementary material for: HIV-1 Transcription Inhibitor 1E7-03 Decreases Nucleophosmin Phosphorylation
Source: Mol Cell Proteomics. 2022 Dec 21;22(2):100488. doi: 10.1016/j.mcpro.2022.100488 (PMC9975258; doi:10.1016/j.mcpro.2022.100488)
Supplement: Supplemental Materials [file mmc1.docx]

**Supplementary Material**

**Method for LC-FT/MS analysis of NPM1 bands at 62 kDa and 250 kDa.**

293T cells were transfected with GFP-tagged NPM1 WT plasmid following the manufacturer’s protocol of Lipofectamine Plus (Invitrogen). At 48 hrs post-transfection, the whole cell extracts were prepared using whole cell lysis buffer (50 mM Tris–HCl, pH 7.5, 0.5 M NaCl, 1% NP-40, 0.1% SDS) supplemented with protease cocktail. The total protein (25 μg) was denatured at 95°C for 10 min and resolved on 10% SDS-PAGE. The 62 kDa and 250 kDa bands were defined and cut into pieces. Gel pieces were crushed into smaller pieces (1 mm^3^), dehydrated with acetonitrile (MP Biochemicals), and rehydrated with 50 mm ammonium bicarbonate for 10 min. Dehydration and rehydration were repeated three times. Reduction and alkylation of the cysteine residues was conducted by treating the samples with 10 mm DTT for 1 h at 60 °C followed by 50 mm iodoacetamide in the dark at room temperature for 30 min. Trypsin Gold (Promega) reconstituted in 50 mm ammonium bicarbonate was added to the gel pieces at a final concentration 10 ng/μL, and the mixture was incubated at 37 °C overnight. Eluted peptides were collected and dried on a SpeedVac concentrator (Thermo Fisher). Samples were reconstituted in 0.1% TFA and purified using C_18_ Zip-tips (Millipore) according to the manufacturer's protocol. Peptides were eluted with 80% acetonitrile containing 0.1% TFA and dried on a SpeedVac.

Nano LC-FT/MS analysis was performed on an UltiMate™ 3000 RSLCnano System coupled to Orbitrap Exploris™ 480 Mass Spectrometer (Thermo Scientific) with the installed Xcalibur software (version 4.4, Thermo Scientific). Purified peptide mixtures were resuspended in 40 μL of water with 0.1% formic acid (v/v). A total of 10 μL of sample was loaded and washed for 5 min on a C_18_-trap column 0.3 × 5 mm, 5 μm, 100 Å,) with a solvent of A:B=98:2 (A, 0.1% formic acid aqueous solution; B, 0.1% formic acid acetonitrile solution) at a constant flow of 300 nL/min. Peptides were transferred onward to a C_18_-packed Aurora series column (25 cm × 75 μm, 1.6 μm, IonOpticks Pty Ltd, Victoria, Australia) and separated with a linear gradient of 5–35 min, 2–25% B, 35–45 min, 25–45% B, 45–50 min, 45-90% B, 50-55 min, 90%B (v/v) at the flow rate of 300 nL/min. Mobile phase A was 0.1% formic acid in water and mobile phase B was 0.1% formic acid in 80% of acetonitrile. The Orbitrap was operated under data-dependent acquisition mode. The spray voltage and capillary temperature were set to 1.8 kV and 325 °C, respectively. Full-scan mass spectra were acquired in Orbitrap resolution 120 000, scan range 350-1200 *m/z*, RF Lens (%) 40 and normalized AGC target (%) 300. Intensity threshold 5×10^3^, charge stage 2-6 and dynamic exclusion 40 s were enabled. Data-dependent MS^2^ scan was carried out with cycle time 3s, isolation window (*m/z*) 1.6, HCD collision energy (%) 35, Orbitrap resolution 15000, maximum injection time 40 ms and AGC target (%) 100.

Nano LC-FT/MS raw data were searched by Proteome Discoverer 2.5 (PD 2.5, Thermo Scientific) using SEQUEST search engine against the Uniprot human database (4/29/2021, 171131 sequences) at a false discovery cut off ≤1%. A maximum of two missed cleavage sites was allowed. The mass tolerance for the precursor ion was set on 10 ppm and for the fragment on 0.02 Da. Phosphorylation of serine, threonine and tyrosine were enabled as dynamic modifications, while carbamidometylation of cysteine was set as fixed modification. Filter settings including minimum peptide length = 6, peptide modification site probability threshold =75 and peptide-spectrum matches with a delta Cn value=0.05 were employed.
