# Supplementary material for: HIV-1 Transcription Inhibitor 1E7-03 Decreases Nucleophosmin Phosphorylation
Source: Mol Cell Proteomics. 2022 Dec 21;22(2):100488. doi: 10.1016/j.mcpro.2022.100488 (PMC9975258; doi:10.1016/j.mcpro.2022.100488)
Supplement: Supplemental Table S9 [file mmc10.docx]

**Supplemental Table S9.** **SIEVE 2.1** **quantification summery for the effect 1E7-03 on phosphorylation profile of the proteins isolated from CEM T cells infected with HIV-1 virus.** CEM T cells, non-infected untreated (i) or treated with 1E7-03 (ii) and HIV-1 infected CEM T cells treated with 1E7-03 (group iv) were compared to CEM T cells infected with HIV-1 and not treated with 1E7-03 that was designated as a reference group iii for SIEVE 2.1 label-free quantification analysis (cutoff > 1.5-fold or <0.66 and *p* value<0.05).

| # | Entrez Gene Name | Symbol | ID | Ratio (group i vs group iii) | Ratio (group ii vs group iii) | Group iii (reference) | Ratio (group iv vs group iii) | Expr p-value | Phosphorylation level changed by 1E7-03 (group iv vs group iv) | Processed methods | Location | Type(s) |
| --- | --- | --- | --- | --- | --- | --- | --- | --- | --- | --- | --- | --- |
| 1 | abl interactor 2 | ABI2 | Q9NYB9 | 1.354 | 2.082 | 1 | 2.59 | 0.003232 | Upregulation | TiO_2_ | Cytoplasm | other |
| 2 | actinin alpha 4 | ACTN4 | O43707 | 1.354 | 2.082 | 1 | 2.59 | 0.003232 | Upregulation | TiO_2_ | Cytoplasm | transcription regulator |
| 3 | AHNAK nucleoprotein | AHNAK | Q09666 | 1.059 | 0.961 | 1 | -2.60 | 0.000060 | Downregulation | TiO_2_ | Nucleus | other |
| 4 | ALG3 alpha-1,3- mannosyltransferase | ALG3 | Q92685 | 1.637 | 1.618 | 1 | 1.73 | 0.003040 | Upregulation | No ENR | Cytoplasm | enzyme |
| 5 | adenosine monophosphate deaminase 3 | AMPD3 | Q01432 | 1.088 | 1.027 | 1 | -2.40 | 0.002980 | Downregulation | No ENR | Cytoplasm | enzyme |
| 6 | ankyrin repeat and LEM domain containing 2 | ANKLE2 | Q86XL3 | 1.550 | 1.304 | 1 | 1.57 | 0.027700 | Upregulation | No ENR | Nucleus | transcription regulator |
| 7 | Rho GTPase activating protein 26 | ARHGAP26 | Q9UNA1 | 1.637 | 1.618 | 1 | 1.73 | 0.003040 | Upregulation | No ENR | Cytoplasm | other |
| 8 | Rho guanine nucleotide exchange factor 16 | ARHGEF16 | Q5VV41 | 1.637 | 1.618 | 1 | 1.73 | 0.018378 | Upregulation | No ENR | Cytoplasm | other |
| 9 | arrestin domain containing 2 | ARRDC2 | Q8TBH0 | 2.186 | 2.011 | 1 | 1.81 | 0.000012 | Upregulation | TiO_2_ | Other | other |
| 10 | argininosuccinate lyase | ASL | P04424 | 1.607 | 1.681 | 1 | 1.84 | 0.014553 | Upregulation | No ENR | Cytoplasm | enzyme |
| 11 | acetylserotonin O-methyltransferase like | ASMTL | O95671 | 0.951 | 1.240 | 1 | 1.59 | 0.022362 | Upregulation | No ENR | Cytoplasm | enzyme |
| 12 | assembly factor for spindle microtubules | ASPM | Q8IZT6 | 0.715 | 1.060 | 1 | 1.61 | 0.010633 | Upregulation | No ENR | Nucleus | other |
| 13 | BAH domain and coiled-coil containing 1 | BAHCC1 | Q9P281 | 1.721 | 2.454 | 1 | 1.72 | 0.000981 | Upregulation | Fe-NTA | Other | other |
| 14 | Bardet-Biedl syndrome 7 | BBS7 | Q8IWZ6 | 1.683 | 2.325 | 1 | 1.54 | 0.018260 | Upregulation | Fe-NTA | Cytoplasm | other |
| 15 | bone morphogenetic protein receptor type 1B | BMPR1B | O00238 | 1.637 | 1.618 | 1 | 1.73 | 0.018378 | Upregulation | No ENR | Plasma Membrane | kinase |
| 16 | B-TFIID TATA-box binding protein associated factor 1 | BTAF1 | O14981 | 1.550 | 1.304 | 1 | 1.57 | 0.027729 | Upregulation | No ENR | Nucleus | transcription regulator |
| 17 | complement C1q like 3 | C1QL3 | L8EC65 | 1.585 | 1.695 | 1 | 1.86 | 0.002937 | Upregulation | TiO_2_ | Extracellular Space | other |
| 18 | C2 calcium dependent domain containing 5 | C2CD5 | Q86YS7 | 1.418 | 0.707 | 1 | 2.58 | 0.000527 | Upregulation | TiO_2_ | Cytoplasm | other |
| 19 | chromosome 2 open reading frame 16 | C2orf16 | A4QMX9 | 1.585 | 1.695 | 1 | 1.86 | 0.002937 | Upregulation | TiO_2_ | Other | other |
| 20 | chromosome 2 open reading frame 42 | C2orf42 | Q9NWW7 | 1.637 | 1.618 | 1 | 1.73 | 0.018378 | Upregulation | No ENR | Nucleus | other |
| 21 | chromosome 8 open reading frame 34 | C8orf34 | Q49A92 | 1.607 | 1.681 | 1 | 1.84 | 0.014553 | Upregulation | No ENR | Other | other |
| 22 | caldesmon 1 | CALD1 | Q05682 | 0.980 | 0.908 | 1 | 0.47 | 0.000007 | Downregulation | Fe-NTA | Cytoplasm | other |
| 23 | caspase 8 associated protein 2 | CASP8AP2 | Q9UKL3 | 1.158 | 0.947 | 1 | 0.43 | 0.000000 | Downregulation | No ENR | Nucleus | transcription regulator |
| 24 | coiled-coil domain containing 171 | CCDC171 | Q6TFL3 | 1.979 | 1.786 | 1 | 1.55 | 0.001763 | Upregulation | TiO_2_ | Other | other |
| 25 | coiled-coil domain containing 7 | CCDC7 | A0A0A6YYA4 | 1.815 | 3.725 | 1 | 2.29 | 0.003714 | Upregulation | Fe-NTA | Other | other |
| 26 | calicin | CCIN | Q8WWB2 | 0.448 | 0.630 | 1 | 0.46 | 0.006882 | Downregulation | TiO_2_ | Cytoplasm | other |
| 27 | CD99 molecule like 2 | CD99L2 | Q8TCZ2 | 1.119 | 2.434 | 1 | 1.55 | 0.002677 | Upregulation | Fe-NTA | Plasma Membrane | other |
| 28 | cell division cycle associated 7 like | CDCA7L | Q96GN5 | 1.019 | 0.892 | 1 | -2.21 | 0.000027 | Downregulation | No ENR | Nucleus | other |
| 29 | ceramide synthase 1 | CERS1 | Q5XG75 | 1.059 | 0.961 | 1 | -2.60 | 0.000000 | Downregulation | TiO_2_ | Cytoplasm | enzyme |
| 30 | chromodomain helicase DNA binding protein 4 | CHD4 | Q14839 | 1.940 | 1.927 | 1 | 1.83 | 0.003114 | Upregulation | TiO_2_ | Nucleus | enzyme |
| 31 | contactin associated protein family member 5 | CNTNAP5 | Q8WYK1 | 1.940 | 1.927 | 1 | 1.83 | 0.000122 | Upregulation | TiO_2_ | Other | other |
| 32 | collagen type VI alpha 2 chain | COL6A2 | C9JH44 | 1.945 | 2.828 | 1 | 1.63 | 0.002195 | Upregulation | Fe-NTA | Extracellular Space | other |
| 33 | CWC22 spliceosome associated protein homolog | CWC22 | Q9HCG8 | 1.815 | 3.725 | 1 | 2.29 | 0.000168 | Upregulation | Fe-NTA | Nucleus | other |
| 34 | decapping enzyme, scavenger | DCPS | Q96C86 | 1.028 | 0.932 | 1 | -2.22 | 0.000555 | Downregulation | No ENR | Nucleus | enzyme |
| 35 | DExD-box helicase 21 | DDX21 | Q9NR30 | 1.940 | 1.927 | 1 | 1.83 | 0.003110 | Upregulation | TiO_2_ | Nucleus | enzyme |
| 36 | DAP3 binding cell death enhancer 1 | DELE1 | Q14154 | 0.920 | 0.832 | 1 | -2.06 | 0.006450 | Downregulation | Fe-NTA | Cytoplasm | other |
| 37 | disco interacting protein 2 homolog C | DIP2C | Q9Y2E4 | 2.199 | 4.012 | 1 | 2.61 | 0.000107 | Upregulation | TiO_2_ | Other | other |
| 38 | DLG associated protein 4 | DLGAP4 | Q9Y2H0 | 1.059 | 0.961 | 1 | -2.60 | 0.000060 | Downregulation | TiO_2_ | Plasma Membrane | other |
| 39 | doublesex and mab-3 related transcription factor 2 | DMRT2 | Q9Y5R5 | 1.059 | 0.961 | 1 | -2.60 | 0.000060 | Downregulation | TiO_2_ | Nucleus | transcription regulator |
| 40 | dynein axonemal heavy chain 7 | DNAH7 | Q8WXX0 | 1.255 | 0.717 | 1 | -2.56 | 0.019900 | Downregulation | No ENR | Cytoplasm | other |
| 41 | DOT1 like histone lysine methyltransferase | DOT1L | H7C2S2 | 1.637 | 1.618 | 1 | 1.73 | 0.018378 | Upregulation | No ENR | Nucleus | phosphatase |
| 42 | dynein regulatory complex subunit 7 | DRC7 | Q8IY82 | 1.637 | 1.618 | 1 | 1.73 | 0.018378 | Upregulation | No ENR | Cytoplasm | other |
| 43 | desmoglein 2 | DSG2 | Q14126 | 1.354 | 2.082 | 1 | 2.59 | 0.003232 | Upregulation | TiO_2_ | Plasma Membrane | other |
| 44 | dystonin | DST | Q6P0N6 | 0.810 | 0.762 | 1 | -2.97 | 0.010200 | Downregulation | No ENR | Plasma Membrane | other |
| 45 | egl-9 family hypoxia inducible factor 1 | EGLN1 | R4SCP5 | 1.585 | 1.695 | 1 | 1.86 | 0.002937 | Upregulation | TiO_2_ | Cytoplasm | enzyme |
| 46 | eukaryotic translation initiation factor 2D | EIF2D | P41214 | 1.607 | 1.681 | 1 | 1.84 | 0.014553 | Upregulation | No ENR | Cytoplasm | transporter |
| 47 | ERCC excision repair 6 like 2 | ERCC6L2 | Q5T890 | 0.758 | 0.893 | 1 | -2.09 | 0.005210 | Downregulation | No ENR | Nucleus | enzyme |
| 48 | ETS transcription factor ERG | ERG | P11308 | 1.059 | 0.961 | 1 | -2.60 | 0.000060 | Downregulation | TiO_2_ | Nucleus | transcription regulator |
| 49 | exocyst complex component 7 | EXOC7 | Q9UPT5 | 1.019 | 0.892 | 1 | -2.21 | 0.000000 | Downregulation | No ENR | Cytoplasm | transporter |
| 50 | coagulation factor VIII | F8 | Q003W2 | 1.059 | 0.961 | 1 | -2.60 | 0.000060 | Downregulation | TiO_2_ | Extracellular Space | other |
| 51 | family with sequence similarity 187 member A | FAM187A | A6NFU0 | 0.758 | 0.893 | 1 | -2.09 | 0.000313 | Downregulation | No ENR | Other | other |
| 52 | FA complementation group I | FANCI | Q9NVI1 | 2.186 | 2.011 | 1 | 1.81 | 0.004310 | Upregulation | TiO_2_ | Nucleus | other |
| 53 | fibrillarin | FBL | Q96BS4 | 1.059 | 0.961 | 1 | -2.60 | 0.000060 | Downregulation | TiO_2_ | Nucleus | enzyme |
| 54 | F-box protein 42 | FBXO42 | Q6P3S6 | 1.354 | 2.082 | 1 | 2.59 | 0.003232 | Upregulation | TiO_2_ | Other | other |
| 55 | FCH and mu domain containing endocytic adaptor 1 | FCHO1 | O14526 | 2.150 | 3.132 | 1 | 2.43 | 0.000000 | Upregulation | TiO_2_ | Plasma Membrane | other |
| 56 | fer-1 like family member 5 | FER1L5 | Q2NNQ7 | 0.606 | 0.681 | 1 | -2.80 | 0.031600 | Downregulation | TiO_2_ | Other | other |
| 57 | FKBP prolyl isomerase 8 | FKBP8 | Q14318 | 1.059 | 0.961 | 1 | -2.60 | 0.000060 | Downregulation | TiO_2_ | Cytoplasm | other |
| 58 | filamin A | FLNA | Q60FE6 | 1.363 | 1.236 | 1 | 1.92 | 0.000603 | Upregulation | TiO_2_ | Cytoplasm | other |
| 59 | formin like 1 | FMNL1 | O95466 | 1.300 | 0.671 | 1 | -2.12 | 0.001020 | Downregulation | TiO_2_ | Cytoplasm | other |
| 60 | fibronectin type III domain containing 1 | FNDC1 | Q4ZHG4 | 1.384 | 2.613 | 1 | 1.82 | 0.001701 | Upregulation | No ENR | Plasma Membrane | other |
| 61 | fibronectin type III domain containing 3A | FNDC3A | Q9Y2H6 | 1.585 | 1.695 | 1 | 1.86 | 0.002937 | Upregulation | TiO_2_ | Cytoplasm | other |
| 62 | fucosyltransferase 10 | FUT10 | Q6P4F1 | 1.015 | 0.617 | 1 | -2.45 | 0.008560 | Downregulation | TiO_2_ | Cytoplasm | enzyme |
| 63 | GRIP and coiled-coil domain containing 2 | GCC2 | Q8IWJ2 | 1.363 | 1.236 | 1 | 1.92 | 0.007474 | Upregulation | TiO_2_ | Cytoplasm | other |
| 64 | GCN1 activator of EIF2AK4 | GCN1 | Q92616 | 0.758 | 0.893 | 1 | -2.09 | 0.005210 | Downregulation | No ENR | Cytoplasm | translation regulator |
| 65 | GINS complex subunit 1 | GINS1 | Q14691 | 1.585 | 1.695 | 1 | 1.86 | 0.002940 | Upregulation | TiO_2_ | Nucleus | other |
| 66 | glycine-N-acyltransferase like 3 | GLYATL3 | Q5SZD4 | 0.531 | 0.074 | 1 | -12.02 | 0.001370 | Downregulation | TiO_2_ | Other | enzyme |
| 67 | germ cell-less 1, spermatogenesis associated | GMCL1 | Q96IK5 | 1.119 | 2.434 | 1 | 1.55 | 0.000092 | Upregulation | Fe-NTA | Nucleus | other |
| 68 | germ cell-less 2, spermatogenesis associated | GMCL2 | Q8NEA9 | 1.119 | 2.434 | 1 | 1.55 | 0.000092 | Upregulation | Fe-NTA | Nucleus | other |
| 69 | GDP-mannose pyrophosphorylase B | GMPPB | Q9Y5P6 | 2.186 | 2.011 | 1 | 1.81 | 0.004315 | Upregulation | TiO_2_ | Cytoplasm | enzyme |
| 70 | glycerol-3-phosphate acyltransferase, mitochondrial | GPAM | Q9HCL2 | 0.982 | 1.043 | 1 | -2.13 | 0.005550 | Downregulation | Fe-NTA | Cytoplasm | enzyme |
| 71 | G protein-coupled receptor class C group 5 member C | GPRC5C | Q9BSP0 | 1.607 | 1.681 | 1 | 1.84 | 0.014553 | Upregulation | No ENR | Plasma Membrane | G-protein coupled receptor |
| 72 | glutamate metabotropic receptor 2 | GRM2 | Q14416 | 0.549 | 1.261 | 1 | 2.42 | 0.044292 | Upregulation | No ENR | Plasma Membrane | G-protein coupled receptor |
| 73 | major histocompatibility complex, class I, B | HLA-B | W0HE80 | 1.354 | 2.082 | 1 | 2.59 | 0.003232 | Upregulation | TiO_2_ | Plasma Membrane | transmembrane receptor |
| 74 | major histocompatibility complex, class I, C | HLA-C | F6KRW2 | 1.354 | 2.082 | 1 | 2.59 | 0.003232 | Upregulation | TiO_2_ | Plasma Membrane | other |
| 75 | heat shock protein 90 alpha family class A member 1 | HSP90AA1 | Q8TBA7 | 1.280 | 0.487 | 1 | -2.64 | 0.000001 | Downregulation | TiO_2_ | Cytoplasm | enzyme |
| 76 | heat shock protein 90 alpha family class B member 1 | HSP90AB1 | Q6PK50 | 1.028 | 0.932 | 1 | -2.22 | 0.000005 | Downregulation | No ENR | Cytoplasm | enzyme |
| 77 | heat shock protein 90 alpha family class B member 2, pseudogene | HSP90AB2P | Q58FF8 | 1.028 | 0.932 | 1 | -2.22 | 0.000005 | Downregulation | No ENR | Cytoplasm | other |
| 78 | inositol hexakisphosphate kinase 2 | IP6K2 | Q9UHH9 | 1.059 | 0.961 | 1 | -2.60 | 0.000000 | Downregulation | TiO_2_ | Cytoplasm | kinase |
| 79 | intersectin 1 | ITSN1 | Q15811 | 1.158 | 0.947 | 1 | -2.31 | 0.001340 | Downregulation | No ENR | Cytoplasm | other |
| 80 | Jupiter microtubule associated homolog 1 | JPT1 | Q9UK76 | 1.107 | 0.795 | 1 | -2.13 | 0.000017 | Downregulation | TiO_2_ | Nucleus | other |
| 81 | LIM homeobox 3 | LHX3 | Q9UBR4 | 1.550 | 1.304 | 1 | 1.57 | 0.027729 | Upregulation | No ENR | Nucleus | transcription regulator |
| 82 | lon peptidase 2, peroxisomal | LONP2 | Q9BU35 | 2.186 | 2.011 | 1 | 1.81 | 0.004315 | Upregulation | TiO_2_ | Cytoplasm | peptidase |
| 83 | leucine rich repeat, Ig-like and transmembrane domains 2 | LRIT2 | A6NDA9 | 2.150 | 3.132 | 1 | 2.43 | 0.000154 | Upregulation | TiO_2_ | Other | other |
| 84 | leucine rich repeat and coiled-coil centrosomal protein 1 | LRRCC1 | Q9C099 | 1.363 | 1.236 | 1 | 1.92 | 0.000603 | Upregulation | TiO_2_ | Nucleus | transporter |
| 85 | LSM14A mRNA processing body assembly factor | LSM14A | Q8ND56 | 2.186 | 2.011 | 1 | 1.81 | 0.004315 | Upregulation | TiO_2_ | Cytoplasm | other |
| 86 | meiosis 1 associated protein | M1AP | Q8TC57 | 1.637 | 1.618 | 1 | 1.73 | 0.018378 | Upregulation | No ENR | Cytoplasm | other |
| 87 | microtubule actin crosslinking factor 1 | MACF1 | Q9UPN3 | 0.977 | 1.955 | 1 | 2.89 | 0.033827 | Upregulation | TiO_2_ | Cytoplasm | other |
| 88 | MAGE family member D2 | MAGED2 | Q9UNF1 | 1.604 | 2.329 | 1 | 1.51 | 0.000488 | Upregulation | TiO_2_ | Plasma Membrane | other |
| 89 | mitogen-activated protein kinase kinase 2 | MAP2K2 | P36507 | 0.866 | 0.830 | 1 | -2.09 | 0.000158 | Downregulation | Fe-NTA | Cytoplasm | kinase |
| 90 | mitogen-activated protein kinase kinase kinase 4 | MAP3K4 | Q9Y6R4 | 1.550 | 1.304 | 1 | 1.57 | 0.027729 | Upregulation | No ENR | Cytoplasm | kinase |
| 91 | microtubule associated protein 4 | MAP4 | Q86Y04 | 1.300 | 0.671 | 1 | -2.12 | 0.000012 | Downregulation | TiO_2_ | Cytoplasm | other |
| 92 | minichromosome maintenance domain containing 2 | MCMDC2 | Q4G0Z9 | 1.702 | 0.742 | 1 | -2.13 | 0.000272 | Downregulation | Fe-NTA | Other | other |
| 93 | mediator of DNA damage checkpoint 1 | MDC1 | Q14676 | 1.300 | 0.671 | 1 | -2.12 | 0.010000 | Downregulation | TiO_2_ | Nucleus | other |
| 94 | mediator complex subunit 15 | MED15 | Q96RN5 | 1.637 | 1.618 | 1 | 1.73 | 0.018378 | Upregulation | No ENR | Nucleus | transcription regulator |
| 95 | mediator complex subunit 23 | MED23 | Q9ULK4 | 1.383 | 0.892 | 1 | -2.56 | 0.003490 | Downregulation | TiO_2_ | Nucleus | transcription regulator |
| 96 | methyltransferase like 1 | METTL1 | Q9UBP6 | 2.186 | 2.011 | 1 | 1.81 | 0.004310 | Upregulation | TiO_2_ | Nucleus | enzyme |
| 97 | monoglyceride lipase | MGLL | Q99685 | 1.059 | 0.961 | 1 | -2.60 | 0.000000 | Downregulation | TiO_2_ | Plasma Membrane | enzyme |
| 98 | membrane integral NOTCH2 associated receptor 1 | MINAR1 | Q9UPX6 | 1.604 | 2.329 | 1 | 1.51 | 0.006653 | Upregulation | TiO_2_ | Plasma Membrane | other |
| 99 | matrix metallopeptidase 2 | MMP2 | P08253 | 1.059 | 0.961 | 1 | -2.60 | 0.000060 | Downregulation | TiO_2_ | Extracellular Space | peptidase |
| 100 | MORN repeat containing 1 | MORN1 | Q5T088 | 1.354 | 2.082 | 1 | 2.59 | 0.003232 | Upregulation | TiO_2_ | Other | other |
| 101 | mitochondrial ribosome associated GTPase 2 | MTG2 | Q9H4K7 | 1.637 | 1.618 | 1 | 1.73 | 0.018378 | Upregulation | No ENR | Cytoplasm | enzyme |
| 102 | major vault protein | MVP | X5DNU0 | 1.604 | 2.329 | 1 | 1.51 | 0.006653 | Upregulation | TiO_2_ | Nucleus | other |
| 103 | MYD88 innate immune signal transduction adaptor | MYD88 | Q99836 | 2.308 | 2.454 | 1 | 2.36 | 0.000624 | Upregulation | TiO_2_ | Plasma Membrane | other |
| 104 | myelin expression factor 2 | MYEF2 | A0A087WUT0 | 1.255 | 0.717 | 1 | -2.56 | 0.003490 | Downregulation | No ENR | Nucleus | transcription regulator |
| 105 | myosin IIIA | MYO3A | Q8NEV4 | 1.585 | 1.695 | 1 | 1.86 | 0.002940 | Upregulation | TiO_2_ | Cytoplasm | kinase |
| 106 | neuron navigator 3 | NAV3 | Q8IVL0 | 1.585 | 1.695 | 1 | 1.86 | 0.000109 | Upregulation | TiO_2_ | Nucleus | other |
| 107 | NAD(P)HX epimerase | NAXE | Q5T3I3 | 1.059 | 0.961 | 1 | -2.60 | 0.000060 | Downregulation | TiO_2_ | Extracellular Space | enzyme |
| 108 | neurofilament heavy | NEFH | P12036 | 0.541 | 1.158 | 1 | 1.85 | 0.004100 | Upregulation | No ENR | Cytoplasm | other |
| 109 | NHL repeat containing E3 ubiquitin protein ligase 1 | NHLRC1 | Q6VVB1 | 1.585 | 1.695 | 1 | 1.86 | 0.002937 | Upregulation | TiO_2_ | Cytoplasm | enzyme |
| 110 | nucleophosmin 1 | NPM1 | A4ZU86 | 0.792 | 0.039 | 1 | -20.15 | 0.000000 | Downregulation | TiO_2_ | Nucleus | transcription regulator |
| 111 | NSE4 homolog A, SMC5-SMC6 complex component | NSMCE4A | Q9NXX6 | 1.637 | 1.618 | 1 | 1.73 | 0.018400 | Upregulation | No ENR | Nucleus | other |
| 112 | 2'-5'-oligoadenylate synthetase 3 | OAS3 | A0A024RBQ5 | 0.709 | 0.734 | 1 | -2.06 | 0.021700 | Downregulation | No ENR | Cytoplasm | enzyme |
| 113 | obscurin, cytoskeletal calmodulin and titin-interacting RhoGEF | OBSCN | Q5VST9 | 1.526 | 1.627 | 1 | 1.65 | 0.034600 | Upregulation | TiO_2_ | Cytoplasm | kinase |
| 114 | outer dense fiber of sperm tails 3 | ODF3 | F8W6Z3 | 1.585 | 1.695 | 1 | 1.86 | 0.002937 | Upregulation | TiO_2_ | Cytoplasm | other |
| 115 | O-GlcNAcase | OGA | O60502 | 1.059 | 0.961 | 1 | -2.60 | 0.000060 | Downregulation | TiO_2_ | Cytoplasm | enzyme |
| 116 | oxysterol binding protein like 5 | OSBPL5 | Q9H0X9 | 1.585 | 1.695 | 1 | 1.86 | 0.000109 | Upregulation | TiO_2_ | Cytoplasm | transporter |
| 117 | phenylalanine hydroxylase | PAH | Q8TEY0 | 2.186 | 2.011 | 1 | 1.81 | 0.004310 | Upregulation | TiO_2_ | Cytoplasm | enzyme |
| 118 | PBX homeobox 4 | PBX4 | Q9BYU1 | 2.506 | 1.441 | 1 | 1.77 | 0.003140 | Upregulation | TiO_2_ | Nucleus | transcription regulator |
| 119 | protocadherin beta 14 | PCDHB14 | Q9Y5E9 | 2.308 | 2.454 | 1 | 2.36 | 0.000624 | Upregulation | TiO_2_ | Plasma Membrane | other |
| 120 | period circadian regulator 1 | PER1 | J3QL46 | 2.308 | 2.454 | 1 | 2.36 | 0.007620 | Upregulation | TiO_2_ | Nucleus | transcription regulator |
| 121 | 6-phosphofructo-2-kinase/fructose-2,6-biphosphatase 3 | PFKFB3 | Q5W015 | 1.721 | 2.454 | 1 | 1.72 | 0.000981 | Upregulation | Fe-NTA | Cytoplasm | kinase |
| 122 | profilin 2 | PFN2 | C9J2N0 | 1.383 | 0.892 | 1 | -2.24 | 0.001390 | Downregulation | TiO_2_ | Cytoplasm | enzyme |
| 123 | peptidoglycan recognition protein 4 | PGLYRP4 | Q96LB8 | 1.550 | 1.304 | 1 | 1.57 | 0.006282 | Upregulation | No ENR | Plasma Membrane | transmembrane receptor |
| 124 | PH domain and leucine rich repeat protein phosphatase 1 | PHLPP1 | O60346 | 0.448 | 0.630 | 1 | -2.19 | 0.006880 | Downregulation | TiO_2_ | Cytoplasm | enzyme |
| 125 | phytanoyl-CoA dioxygenase domain containing 1 | PHYHD1 | X6RJK6 | 1.637 | 1.618 | 1 | 1.73 | 0.018378 | Upregulation | No ENR | Other | other |
| 126 | polycystin family receptor for egg jelly | PKDREJ | Q9NTG1 | 1.604 | 2.329 | 1 | 1.51 | 0.000488 | Upregulation | TiO_2_ | Plasma Membrane | ion channel |
| 127 | plasminogen | PLG | Q5TEH5 | 0.549 | 1.261 | 1 | 2.42 | 0.014192 | Upregulation | No ENR | Extracellular Space | peptidase |
| 128 | pregnancy up-regulated nonubiquitous CaM kinase | PNCK | Q6P2M8 | 2.150 | 3.132 | 1 | 2.43 | 0.000154 | Upregulation | TiO_2_ | Other | kinase |
| 129 | DNA polymerase theta | POLQ | D3DN91 | 1.550 | 1.304 | 1 | 1.57 | 0.027729 | Upregulation | No ENR | Nucleus | enzyme |
| 130 | protein O-mannosyltransferase 2 | POMT2 | Q9UKY4 | 1.637 | 1.618 | 1 | 1.73 | 0.003037 | Upregulation | No ENR | Cytoplasm | enzyme |
| 131 | peter pan homolog | PPAN | Q9NQ55 | 1.550 | 1.304 | 1 | 1.57 | 0.006282 | Upregulation | No ENR | Nucleus | other |
| 132 | PPAN-P2RY11 readthrough | PPAN-P2RY11 | A0A0B4J1V8 | 1.550 | 1.304 | 1 | 1.57 | 0.006282 | Upregulation | No ENR | Other | other |
| 133 | protein phosphatase 1 regulatory subunit 26 | PPP1R26 | Q5T8A7 | 0.549 | 1.261 | 1 | 2.42 | 0.044292 | Upregulation | No ENR | Nucleus | other |
| 134 | protein phosphatase 1 regulatory subunit 42 | PPP1R42 | Q7Z4L9 | 1.059 | 0.961 | 1 | 0.38 | 0.000060 | Downregulation | TiO_2_ | Cytoplasm | other |
| 135 | PRELI domain containing 3B | PRELID3B | Q9Y3B1 | 1.354 | 2.082 | 1 | 2.59 | 0.003232 | Upregulation | TiO_2_ | Cytoplasm | other |
| 136 | protein kinase Y-linked (pseudogene) | PRKY | O43930 | 1.028 | 0.932 | 1 | 0.45 | 0.000555 | Downregulation | No ENR | Other | kinase |
| 137 | protein tyrosine phosphatase receptor type S | PTPRS | Q59FX6 | 1.550 | 1.304 | 1 | 1.57 | 0.027729 | Upregulation | No ENR | Plasma Membrane | phosphatase |
| 138 | glycogen phosphorylase, muscle associated | PYGM | P11217 | 1.059 | 0.961 | 1 | 0.38 | 0.000060 | Downregulation | TiO_2_ | Cytoplasm | enzyme |
| 139 | QKI, KH domain containing RNA binding | QKI | Q8WY44 | 1.945 | 2.828 | 1 | 1.63 | 0.002195 | Upregulation | Fe-NTA | Nucleus | other |
| 140 | Ral GTPase activating protein catalytic subunit alpha 1 | RALGAPA1 | L8EAR6 | 0.758 | 0.893 | 1 | -2.09 | 0.000313 | Downregulation | No ENR | Cytoplasm | other |
| 141 | arginyl-tRNA synthetase 2, mitochondrial | RARS2 | Q5T160 | 1.059 | 0.961 | 1 | -2.60 | 0.000000 | Downregulation | TiO_2_ | Cytoplasm | enzyme |
| 142 | replication factor C subunit 4 | RFC4 | P35249 | 1.383 | 0.892 | 1 | -2.24 | 0.001390 | Downregulation | TiO_2_ | Nucleus | other |
| 143 | regulator of G protein signaling 12 | RGS12 | Q56A82 | 1.059 | 0.961 | 1 | -2.60 | 0.000000 | Downregulation | TiO_2_ | Nucleus | enzyme |
| 144 | regulator of G protein signaling like 1 | RGSL1 | H3BU64 | 1.059 | 0.961 | 1 | -2.60 | 0.000000 | Downregulation | TiO_2_ | Other | other |
| 145 | ring finger protein 213 | RNF213 | Q63HN8 | 1.017 | 0.892 | 1 | -2.41 | 0.000493 | Downregulation | TiO_2_ | Cytoplasm | enzyme |
| 146 | regulator of solute carriers 1 | RSC1A1 | Q92681 | 0.758 | 0.893 | 1 | -2.09 | 0.005210 | Downregulation | No ENR | Nucleus | other |
| 147 | rotatin | RTTN | Q86VV8 | 0.977 | 0.936 | 1 | -2.06 | 0.000043 | Downregulation | Fe-NTA | Cytoplasm | other |
| 148 | selenocysteine lyase | SCLY | H7C277 | 1.945 | 2.828 | 1 | 1.63 | 0.002195 | Upregulation | Fe-NTA | Cytoplasm | enzyme |
| 149 | sodium channel epithelial 1 subunit alpha | SCNN1A | P37088 | 0.810 | 0.762 | 1 | -2.97 | 0.036600 | Downregulation | No ENR | Plasma Membrane | ion channel |
| 150 | splicing factor 3b subunit 1 | SF3B1 | Q7Z497 | 1.354 | 2.082 | 1 | 2.59 | 0.003230 | Upregulation | TiO_2_ | Nucleus | other |
| 151 | N-sulfoglucosamine sulfohydrolase | SGSH | I3L0M2 | 2.186 | 2.011 | 1 | 1.81 | 0.004315 | Upregulation | TiO_2_ | Cytoplasm | enzyme |
| 152 | solute carrier family 6 member 15 | SLC6A15 | Q9H2J7 | 0.966 | 1.182 | 1 | 1.92 | 0.000000 | Upregulation | TiO_2_ | Plasma Membrane | transporter |
| 153 | SMAD family member 7 | SMAD7 | O15105 | 1.637 | 1.618 | 1 | 1.73 | 0.018378 | Upregulation | No ENR | Nucleus | transcription regulator |
| 154 | sorting nexin 18 | SNX18 | Q96RF0 | 1.495 | 1.371 | 1 | 1.54 | 0.000847 | Upregulation | No ENR | Cytoplasm | transporter |
| 155 | SOGA family member 3 | SOGA3 | Q5TF21 | 1.059 | 0.961 | 1 | -2.60 | 0.000000 | Downregulation | TiO_2_ | Other | other |
| 156 | SP140 nuclear body protein | SP140 | Q13342 | 1.604 | 2.329 | 1 | 1.51 | 0.006653 | Upregulation | TiO_2_ | Nucleus | transcription regulator |
| 157 | SPOUT domain containing methyltransferase 1 | SPOUT1 | R4GNG4 | 1.585 | 1.695 | 1 | 1.86 | 0.000109 | Upregulation | TiO_2_ | Nucleus | other |
| 158 | splA/ryanodine receptor domain and SOCS box containing 3 | SPSB3 | Q96LS6 | 1.034 | 0.632 | 1 | -2.14 | 0.002410 | Downregulation | No ENR | Cytoplasm | other |
| 159 | serine/arginine repetitive matrix 5 | SRRM5 | Q4G0Z0 | 1.354 | 2.082 | 1 | 2.59 | 0.003232 | Upregulation | TiO_2_ | Other | other |
| 160 | serine and arginine rich splicing factor 11 | SRSF11 | Q5T757 | 1.119 | 2.434 | 1 | 1.55 | 0.000000 | Upregulation | Fe-NTA | Nucleus | other |
| 161 | signal transducer and activator of transcription 3 | STAT3 | P40763 | 1.119 | 2.434 | 1 | 1.55 | 0.000000 | Upregulation | Fe-NTA | Nucleus | transcription regulator |
| 162 | syntaxin 18 | STX18 | Q9P2W9 | 2.308 | 2.454 | 1 | 2.36 | 0.007617 | Upregulation | TiO_2_ | Cytoplasm | transporter |
| 163 | spectrin repeat containing nuclear envelope protein 2 | SYNE2 | Q8WXH0 | 1.094 | 1.771 | 1 | 1.55 | 0.000204 | Upregulation | Fe-NTA | Nucleus | other |
| 164 | TATA-box binding protein associated factor 4 | TAF4 | V9GY14 | 1.637 | 1.618 | 1 | 1.73 | 0.000527 | Upregulation | No ENR | Nucleus | transcription regulator |
| 165 | Tax1 binding protein 1 | TAX1BP1 | Q86VP1 | 1.059 | 0.961 | 1 | -2.60 | 0.000060 | Downregulation | TiO_2_ | Cytoplasm | other |
| 166 | TBC1 domain family member 29, pseudogene | TBC1D29P | Q9UFV1 | 2.308 | 2.454 | 1 | 2.36 | 0.007617 | Upregulation | TiO_2_ | Other | other |
| 167 | TBC1 domain family member 32 | TBC1D32 | Q96NH3 | 1.088 | 1.027 | 1 | -2.40 | 0.036800 | Downregulation | No ENR | Other | other |
| 168 | transcription elongation factor A2 | TCEA2 | Q86VL0 | 0.977 | 1.955 | 1 | 2.89 | 0.002430 | Upregulation | TiO_2_ | Nucleus | transcription regulator |
| 169 | transcription elongation factor A N-terminal and central domain containing 2 | TCEANC2 | X6R7X0 | 0.971 | 0.867 | 1 | -2.13 | 0.000308 | Downregulation | Fe-NTA | Other | other |
| 170 | transcription elongation regulator 1 | TCERG1 | O14776 | 0.448 | 0.630 | 1 | -2.19 | 0.006880 | Downregulation | TiO_2_ | Nucleus | transcription regulator |
| 171 | transforming growth factor beta 2 | TGFB2 | Q59EG9 | 0.531 | 0.074 | 1 | -12.02 | 0.001370 | Downregulation | TiO_2_ | Extracellular Space | growth factor |
| 172 | trans-golgi network protein 2 | TGOLN2 | O43493 | 1.059 | 0.961 | 1 | -2.60 | 0.000060 | Downregulation | TiO_2_ | Cytoplasm | other |
| 173 | trinucleotide repeat containing 18 | TNRC18 | O15417 | 1.721 | 2.454 | 1 | 1.72 | 0.000981 | Upregulation | Fe-NTA | Nucleus | other |
| 174 | TOG array regulator of axonemal microtubules 1 | TOGARAM1 | Q9Y4F4 | 1.550 | 1.304 | 1 | 1.57 | 0.027700 | Upregulation | No ENR | Extracellular Space | other |
| 175 | tumor protein p53 | TP53 | Q1MSW8 | 1.059 | 0.961 | 1 | -2.60 | 0.000000 | Downregulation | TiO_2_ | Nucleus | transcription regulator |
| 176 | TRAF3 interacting protein 3 | TRAF3IP3 | Q9Y228 | 2.186 | 2.011 | 1 | 1.81 | 0.004310 | Upregulation | TiO_2_ | Other | other |
| 177 | T cell receptor delta variable 1 | TRDV1 | A0A087X2F4 | 0.549 | 1.261 | 1 | 2.42 | 0.044300 | Upregulation | No ENR | Other | other |
| 178 | trio Rho guanine nucleotide exchange factor | TRIO | O75962 | 1.585 | 1.695 | 1 | 1.86 | 0.002940 | Upregulation | TiO_2_ | Cytoplasm | kinase |
| 179 | transcriptional repressor GATA binding 1 | TRPS1 | Q9UHF7 | 1.815 | 3.725 | 1 | 2.29 | 0.000168 | Upregulation | Fe-NTA | Nucleus | transcription regulator |
| 180 | transformation/transcription domain associated protein | TRRAP | Q9Y4A5 | 0.951 | 1.240 | 1 | 1.59 | 0.022400 | Upregulation | No ENR | Nucleus | transcription regulator |
| 181 | tetratricopeptide repeat domain 21A | TTC21A | Q8NDW8 | 1.495 | 1.371 | 1 | 1.54 | 0.000847 | Upregulation | No ENR | Extracellular Space | other |
| 182 | titin | TTN | Q8WZ42 | 1.815 | 3.725 | 1 | -2.32 | 0.000182 | Upregulation | Fe-NTA | Cytoplasm | kinase |
| 183 | tubulin alpha 1b | TUBA1B | P68363 | 1.637 | 1.618 | 1 | 1.73 | 0.003040 | Upregulation | No ENR | Cytoplasm | other |
| 184 | tubulin alpha 1c | TUBA1C | Q8N532 | 1.607 | 1.681 | 1 | 1.84 | 0.014600 | Upregulation | No ENR | Cytoplasm | other |
| 185 | tubulin alpha 4a | TUBA4A | P68366 | 1.637 | 1.618 | 1 | 1.73 | 0.003040 | Upregulation | No ENR | Cytoplasm | other |
| 186 | tubulin alpha 4b | TUBA4B | Q9H853 | 1.637 | 1.618 | 1 | 1.73 | 0.003040 | Upregulation | No ENR | Cytoplasm | other |
| 187 | tubulin alpha 8 | TUBA8 | Q9NY65 | 1.637 | 1.618 | 1 | 1.73 | 0.003040 | Upregulation | No ENR | Cytoplasm | enzyme |
| 188 | tubulin beta class I | TUBB | Q9BUU9 | 0.448 | 0.630 | 1 | -2.19 | 0.000519 | Downregulation | TiO_2_ | Cytoplasm | other |
| 189 | tubulin beta 4A class IVa | TUBB4A | P04350 | 0.448 | 0.630 | 1 | -2.19 | 0.000519 | Downregulation | TiO_2_ | Cytoplasm | other |
| 190 | tubulin beta 4B class IVb | TUBB4B | P68371 | 0.448 | 0.630 | 1 | -2.19 | 0.000519 | Downregulation | TiO_2_ | Cytoplasm | other |
| 191 | thymidine phosphorylase | TYMP | B2RBL3 | 1.637 | 1.618 | 1 | 1.73 | 0.018400 | Upregulation | No ENR | Extracellular Space | growth factor |
| 192 | ubiquitin specific peptidase 13 | USP13 | Q92995 | 1.019 | 0.892 | 1 | -2.21 | 0.001390 | Downregulation | No ENR | Cytoplasm | peptidase |
| 193 | ubiquitin specific peptidase 47 | USP47 | Q96K76 | 1.550 | 1.304 | 1 | 1.57 | 0.027700 | Upregulation | No ENR | Cytoplasm | peptidase |
| 194 | ubiquitously transcribed tetratricopeptide repeat containing, Y-linked | UTY | E1NZ79 | 1.637 | 1.618 | 1 | 1.73 | 0.000094 | Upregulation | No ENR | Nucleus | enzyme |
| 195 | versican | VCAN | Q59FG9 | 1.088 | 1.027 | 1 | -2.40 | 0.000269 | Downregulation | No ENR | Extracellular Space | other |
| 196 | WD repeat and FYVE domain containing 3 | WDFY3 | Q8IZQ1 | 1.815 | 3.725 | 1 | 2.29 | 0.000168 | Upregulation | Fe-NTA | Cytoplasm | enzyme |
| 197 | WD repeat domain 59 | WDR59 | Q6PJI9 | 1.158 | 0.947 | 1 | -2.31 | 0.000026 | Downregulation | No ENR | Cytoplasm | transporter |
| 198 | YKT6 v-SNARE homolog | YKT6 | O15498 | 0.790 | 0.944 | 1 | -2.70 | 0.000220 | Downregulation | Fe-NTA | Cytoplasm | enzyme |
| 199 | zinc finger protein 197 | ZNF197 | Q4G1C8 | 2.506 | 1.441 | 1 | 1.77 | 0.018700 | Upregulation | TiO_2_ | Nucleus | transcription regulator |
| 200 | zinc finger protein 226 | ZNF226 | Q9NYT6 | 1.585 | 1.695 | 1 | 1.86 | 0.002940 | Upregulation | TiO_2_ | Nucleus | transcription regulator |
| 201 | zinc finger protein 474 | ZNF474 | Q6S9Z5 | 1.637 | 1.618 | 1 | 1.73 | 0.003040 | Upregulation | No ENR | Other | other |
| 202 | zinc finger protein 585B | ZNF585B | K7EL12 | 1.359 | 1.440 | 1 | 1.74 | 0.038500 | Upregulation | No ENR | Nucleus | other |
| 203 | zinc finger protein 831 | ZNF831 | Q5JPB2 | 1.604 | 2.329 | 1 | 1.51 | 0.006650 | Upregulation | TiO_2_ | Other | other |
| 204 | zinc finger protein 90 | ZNF90 | Q03938 | 1.637 | 1.618 | 1 | 1.73 | 0.018400 | Upregulation | No ENR | Nucleus | transcription regulator |
| 205 | zinc finger protein 99 | ZNF99 | A8MXY4 | 0.844 | 0.399 | 1 | -2.11 | 0.008820 | Downregulation | TiO_2_ | Other | other |
| 206 | zinc finger and SCAN domain containing 18 | ZSCAN18 | A0A0C4DG78 | 1.637 | 1.618 | 1 | 1.73 | 0.018400 | Upregulation | No ENR | Other | other |
