# Supplementary material for: HIV-1 Transcription Inhibitor 1E7-03 Decreases Nucleophosmin Phosphorylation
Source: Mol Cell Proteomics. 2022 Dec 21;22(2):100488. doi: 10.1016/j.mcpro.2022.100488 (PMC9975258; doi:10.1016/j.mcpro.2022.100488)
Supplement: Supplemental Table S10 [file mmc11.docx]

Supplemental Table S10. Pathways identified by Ingenuity canonical pathway analysis for proteins which phosphorylation was affected by 1E7-03.

| Ingenuity Canonical Pathways | -log(p-value) | Ratio | Z-score | Molecules |
| --- | --- | --- | --- | --- |
| Coronavirus Replication Pathway | 8.34 | 0.178 | 0.707 | TUBA1B,TUBA1C,TUBA4A,TUBA4B,TUBA8,TUBB,TUBB4A,TUBB4B |
| Breast Cancer Regulation by Stathmin1 | 3.65 | 0.0253 | -0.258 | ARHGEF16,GPRC5C,GRM2,MAP2K2,MMP2,STAT3,TGFB2,TP53,TUBA1B,TUBA1C,TUBA4A,TUBA8,TUBB,TUBB4A,TUBB4B |
| Sirtuin Signaling Pathway | 3.59 | 0.0341 | 1.265 | DOT1L,PFKFB3,SCNN1A,STAT3,TP53,TUBA1B,TUBA1C,TUBA4A,TUBA4B,TUBA8 |
| Role of PKR in Interferon Induction and Antiviral Response | 2.91 | 0.0441 | 1.633 | HSP90AA1,HSP90AB1,MYD88,NPM1,STAT3,TP53 |
| HIF1α Signaling | 2.63 | 0.0337 | -1.134 | EGLN1,HSP90AA1,MAP2K2,MMP2,STAT3,TGFB2,TP53 |
| Tumor Microenvironment Pathway | 2.32 | 0.0335 | -1.633 | HLA-B,HLA-C,MAP2K2,MMP2,STAT3,TGFB2 |
| Actin Cytoskeleton Signaling | 2.25 | 0.0287 | -0.447 | ABI2,ACTN4,FLNA,MAP2K2,PFN2,TRIO,TTN |
| TGF-β Signaling | 2.01 | 0.0417 | -1 | BMPR1B,MAP2K2,SMAD7,TGFB2 |
| Telomerase Signaling | 1.84 | 0.037 | -1 | HSP90AA1,HSP90AB1,MAP2K2,TP53 |
| NGF Signaling | 1.68 | 0.0333 | 0 | MAP2K2,MAP3K4,TP53,TRIO |
| Acute Phase Response Signaling | 1.64 | 0.027 | 0.447 | F8,MAP2K2,MYD88,PLG,STAT3 |
| MicroRNA Biogenesis Signaling Pathway | 1.62 | 0.0267 | -1.342 | HSP90AA1,HSP90AB1,QKI,TGFB2,TP53 |
| Estrogen Receptor Signaling | 1.57 | 0.0196 | 0 | HSP90AA1,HSP90AB1,MAP2K2,MED15,MED23,MMP2,TP53,TRRAP |
| PPARα/RXRα Activation | 1.55 | 0.0256 | 2 | HSP90AA1,HSP90AB1,MAP2K2,MED23,TGFB2 |
| Natural Killer Cell Signaling | 1.53 | 0.0253 | -0.447 | HLA-B,HLA-C,MAP2K2,MAP3K4,MYD88 |
| Pulmonary Healing Signaling Pathway | 1.52 | 0.0251 | 1.342 | BMPR1B,MMP2,MYD88,STAT3,TGFB2 |
| STAT3 Pathway | 1.52 | 0.0296 | 0 | BMPR1B,MAP2K2,STAT3,TGFB2 |
| ID1 Signaling Pathway | 1.51 | 0.0249 | 0.447 | BMPR1B,MMP2,STAT3,TGFB2,TP53 |
| Integrin Signaling | 1.42 | 0.0236 | -1.342 | ACTN4,ARHGAP26,MAP2K2,PFN2,TTN |
| Aryl Hydrocarbon Receptor Signaling | 1.3 | 0.0252 | 2 | HSP90AA1,HSP90AB1,TGFB2,TP53 |
| Pulmonary Fibrosis Idiopathic Signaling Pathway | 1.18 | 0.0184 | 0 | COL6A2,MMP2,PLG,STAT3,TGFB2,TP53 |
| IL-17 Signaling | 1.1 | 0.0214 | -2 | HSP90AA1,HSP90AB1,MMP2,TGFB2 |
| Colorectal Cancer Metastasis Signaling | 1.06 | 0.0185 | 0 | MAP2K2,MMP2,STAT3,TGFB2,TP53 |
| Leukocyte Extravasation Signaling | 1.06 | 0.0207 | 0 | ACTN4,MAP2K2,MAP3K4,MMP2 |
| Regulation Of The Epithelial Mesenchymal Transition By Growth Factors Pathway | 1.06 | 0.0208 | -1 | MAP2K2,MMP2,STAT3,TGFB2 |
| PI3K/AKT Signaling | 1.01 | 0.02 | -2 | HSP90AA1,HSP90AB1,MAP2K2,TP53 |
| Coronavirus Pathogenesis Pathway | 0.991 | 0.0196 | 0 | NPM1,OAS3,STAT3,TP53 |
| CLEAR Signaling Pathway | 0.987 | 0.0175 | 0.447 | BMPR1B,SGSH,TGFB2,TP53,WDR59 |
| Autophagy | 0.924 | 0.0185 | 0 | MAP2K2,MYD88,TGFB2,TP53 |
| Multiple Sclerosis Signaling Pathway | 0.893 | 0.018 | 0 | HLA-B,HLA-C,RNF213,TGFB2 |
| HER-2 Signaling in Breast Cancer | 0.866 | 0.0176 | 0 | MAP2K2,MMP2,STAT3,TP53 |
| Protein Kinase A Signaling | 0.833 | 0.0146 | -2 | FLNA,MAP2K2,PTPRS,PYGM,TGFB2,TTN |
| cAMP-mediated signaling | 0.83 | 0.017 | 1 | GRM2,MAP2K2,RGS12,STAT3 |
| Role Of Osteoblasts In Rheumatoid Arthritis Signaling Pathway | 0.807 | 0.0167 | -1 | BMPR1B,MMP2,STAT3,TGFB2 |
| Hepatic Fibrosis Signaling Pathway | 0.788 | 0.0142 | -0.816 | MAP2K2,MYD88,SMAD7,STAT3,TGFB2,TTN |
| Senescence Pathway | 0.582 | 0.0134 | -1 | MAP2K2,SMAD7,TGFB2,TP53 |
| Neuroinflammation Signaling Pathway | 0.529 | 0.0126 | 1 | HLA-B,HLA-C,MYD88,TGFB2 |
| Pathogen Induced Cytokine Storm Signaling Pathway | 0.394 | 0.0108 | 1 | COL6A2,MYD88,STAT3,TGFB2 |
| G-Protein Coupled Receptor Signaling | 0.388 | 0.00997 | 1.134 | GPRC5C,GRM2,MAP2K2,MAP3K4,RGS12,STAT3,TTN |
