# Supplementary material for: HIV-1 Transcription Inhibitor 1E7-03 Decreases Nucleophosmin Phosphorylation
Source: Mol Cell Proteomics. 2022 Dec 21;22(2):100488. doi: 10.1016/j.mcpro.2022.100488 (PMC9975258; doi:10.1016/j.mcpro.2022.100488)
Supplement: Supplemental Table S11 [file mmc12.docx]

**Supplemental Table S11. 1E7-03 treatment of CEM T cells affected expression of 50 proteins.**

Expression of 50 proteins out of 5311 detected human proteins was upregulated or downregulated by 1E7-03.

| # | Entrez Gene Name | Symbol | Expr Fold Change | Expr p-value | Location | Type(s) |
| --- | --- | --- | --- | --- | --- | --- |
| 1 | ankyrin repeat domain 11 | ANKRD11 | -1.792 | 0.0106 | Nucleus | other |
| 2 | Rho GTPase activating protein 8 | ARHGAP8/PRR5-ARHGAP8 | 1.612 | 0.00902 | Cytoplasm | other |
| 3 | arrestin domain containing 4 | ARRDC4 | 1.669 | 0.0127 | Plasma Membrane | other |
| 4 | autophagy related 16 like 1 | ATG16L1 | 1.664 | 0.00556 | Cytoplasm | enzyme |
| 5 | chromosome 3 open reading frame 33 | C3orf33 | -1.894 | 0.0401 | Extracellular Space | other |
| 6 | chaperonin containing TCP1 subunit 7 | CCT7 | -2.058 | 0.00502 | Cytoplasm | other |
| 7 | DNA cross-link repair 1C | DCLRE1C | 1.701 | 0.00469 | Nucleus | enzyme |
| 8 | EBF transcription factor 1 | EBF1 | -1.733 | 0.0135 | Nucleus | transcription regulator |
| 9 | eukaryotic translation initiation factor 3 subunit F | EIF3F | -1.855 | 0.037 | Cytoplasm | translation regulator |
| 10 | fatty acid synthase | FASN | -1.961 | 0.00357 | Cytoplasm | enzyme |
| 11 | fidgetin like 1 | FIGNL1 | -1.597 | 0.0278 | Nucleus | enzyme |
| 12 | H3.2 histone (putative) | H3-2 | 1.516 | 0.0216 | Nucleus | other |
| 13 | H3.3 histone A | H3-3A/H3-3B | 1.516 | 0.0216 | Nucleus | other |
| 14 | H3.4 histone | H3-4 | 1.516 | 0.0216 | Nucleus | other |
| 15 | H3.5 histone | H3-5 | 1.516 | 0.0216 | Other | other |
| 16 | helicase with zinc finger 2 | HELZ2 | -2.066 | 0.00178 | Nucleus | transcription regulator |
| 17 | major histocompatibility complex, class I, G | HLA-G | -2.062 | 0.000458 | Plasma Membrane | other |
| 18 | heat shock protein 90 alpha family class A member 1 | HSP90AA1 | -1.733 | 0.00893 | Cytoplasm | enzyme |
| 19 | heat shock protein 90 alpha family class A member 5, pseudogene | HSP90AA5P | -2.049 | 0.00165 | Other | other |
| 20 | heat shock protein family A (Hsp70) member 8 | HSPA8 | -1.56 | 0.037 | Cytoplasm | enzyme |
| 21 | heat shock protein family D (Hsp60) member 1 | HSPD1 | -1.838 | 0.00568 | Cytoplasm | enzyme |
| 22 | KIAA0232 | KIAA0232 | -1.582 | 0.0411 | Extracellular Space | other |
| 23 | klotho beta | KLB | 1.826 | 0.00117 | Plasma Membrane | enzyme |
| 24 | kynurenine 3-monooxygenase | KMO | 2.478 | 0.00000159 | Cytoplasm | enzyme |
| 25 | LSM7 homolog, U6 small nuclear RNA and mRNA degradation associated | LSM7 | -1.862 | 0.00782 | Nucleus | other |
| 26 | MORC family CW-type zinc finger 4 | MORC4 | 2.238 | 0.0000185 | Nucleus | other |
| 27 | N-acetyltransferase 8B (putative, gene/pseudogene) | NAT8B | -2.364 | 0.0146 | Cytoplasm | enzyme |
| 28 | NIPA like domain containing 4 | NIPAL4 | 1.702 | 0.0039 | Other | other |
| 29 | NOP9 nucleolar protein | NOP9 | 2.478 | 0.00000159 | Nucleus | other |
| 30 | nuclear receptor subfamily 1 group D member 1 | NR1D1 | -1.555 | 0.0384 | Nucleus | ligand-dependent nuclear receptor |
| 31 | olfactory receptor family 10 subfamily AG member 1 | OR10AG1 | 1.631 | 0.00756 | Plasma Membrane | G-protein coupled receptor |
| 32 | OTU deubiquitinase, ubiquitin aldehyde binding 1 | OTUB1 | 1.512 | 0.0223 | Cytoplasm | enzyme |
| 33 | procollagen C-endopeptidase enhancer 2 | PCOLCE2 | -1.686 | 0.0238 | Extracellular Space | other |
| 34 | pyridoxal kinase | PDXK | -2.364 | 0.0146 | Cytoplasm | kinase |
| 35 | proteasome 26S subunit, non-ATPase 6 | PSMD6 | -1.548 | 0.041 | Cytoplasm | enzyme |
| 36 | RAB5B, member RAS oncogene family | RAB5B | 1.81 | 0.00138 | Cytoplasm | enzyme |
| 37 | scratch family transcriptional repressor 1 | SCRT1 | -1.634 | 0.0256 | Nucleus | transcription regulator |
| 38 | SH2 domain containing 7 | SH2D7 | 2.478 | 0.00000159 | Other | other |
| 39 | SET and MYND domain containing 3 | SMYD3 | -1.597 | 0.0274 | Nucleus | enzyme |
| 40 | ST3 beta-galactoside alpha-2,3-sialyltransferase 3 | ST3GAL3 | -2.653 | 0.0000746 | Cytoplasm | enzyme |
| 41 | TBC1 domain family member 32 | TBC1D32 | -1.645 | 0.0213 | Other | other |
| 42 | tubulin folding cofactor E like | TBCEL | -1.686 | 0.0238 | Other | other |
| 43 | DNA topoisomerase II alpha | TOP2A | -1.779 | 0.00854 | Nucleus | enzyme |
| 44 | thioredoxin domain containing 9 | TXNDC9 | 1.767 | 0.00249 | Cytoplasm | other |
| 45 | UFM1 specific peptidase 2 | UFSP2 | -1.582 | 0.0312 | Other | enzyme |
| 46 | WD repeat and FYVE domain containing 3 | WDFY3 | -1.792 | 0.0106 | Cytoplasm | enzyme |
| 47 | zinc finger CCCH-type containing 18 | ZC3H18 | 1.789 | 0.00155 | Nucleus | other |
| 48 | zinc finger protein 418 | ZNF418 | -3.003 | 0.000518 | Nucleus | transcription regulator |
| 49 | zinc finger protein 585B | ZNF585B | 1.664 | 0.00556 | Nucleus | other |
| 50 | zinc finger protein 806 | ZNF806 | 1.514 | 0.0219 | Other | other |
