# Supplementary material for: HIV-1 Transcription Inhibitor 1E7-03 Decreases Nucleophosmin Phosphorylation
Source: Mol Cell Proteomics. 2022 Dec 21;22(2):100488. doi: 10.1016/j.mcpro.2022.100488 (PMC9975258; doi:10.1016/j.mcpro.2022.100488)
Supplement: Supplemental Table S12 [file mmc13.docx]

Supplemental Table S12. Pathways identified by Ingenuity canonical pathway analysis for proteins which expression levels were affected by 1E7-03.

| Ingenuity Canonical Pathways | -log(p-value) | Ratio | Z-score | Molecules |
| --- | --- | --- | --- | --- |
| Protein Ubiquitination Pathway | 3.8 | 0.0182 |  | HLA-G, HSP90AA1, HSPA8, HSPD1, PSMD6 |
| BAG2 Signaling Pathway | 3.27 | 0.0357 |  | HSP90AA1, HSPA8, PSMD6 |
| Immunogenic Cell Death Signaling Pathway | 3.19 | 0.0333 |  | ATG16L1, HSP90AA1, HSPA8 |
| Fatty Acid Biosynthesis Initiation II | 2.42 | 0.5 |  | FASN |
| Aldosterone Signaling in Epithelial Cells | 2.41 | 0.0179 |  | HSP90AA1, HSPA8, HSPD1 |
| Palmitate Biosynthesis I (Animals) | 2.25 | 0.333 |  | FASN |
| PPARα/RXRα Activation | 2.23 | 0.0154 |  | FASN, HELZ2, HSP90AA1 |
| Caveolar-mediated Endocytosis Signaling | 2.06 | 0.0267 |  | HLA-G, RAB5B |
| Tryptophan Degradation to 2-amino-3-carboxymuconate Semialdehyde | 1.95 | 0.167 |  | KMO |
| Glucocorticoid Receptor Signaling | 1.63 | 0.00686 |  | H3-4, HLA-G, HSP90AA1, HSPA8 |
| NAD biosynthesis II (from tryptophan) | 1.62 | 0.0769 |  | KMO |
| Oleate Biosynthesis II (Animals) | 1.62 | 0.0769 |  | UFSP2 |
| DNA Double-Strand Break Repair by Non-Homologous End Joining | 1.59 | 0.0714 |  | DCLRE1C |
| Adipogenesis pathway | 1.57 | 0.0148 |  | EBF1, NR1D1 |
| Role of PKR in Interferon Induction and Antiviral Response | 1.57 | 0.0147 |  | HSP90AA1, HSPA8 |
| eNOS Signaling | 1.46 | 0.0128 |  | HSP90AA1, HSPA8 |
| Phagosome Maturation | 1.45 | 0.0127 |  | HLA-G, RAB5B |
| Androgen Signaling | 1.39 | 0.0118 |  | H3-4, HSP90AA1 |
| Tryptophan Degradation III (Eukaryotic) | 1.32 | 0.0385 |  | KMO |
| MicroRNA Biogenesis Signaling Pathway | 1.32 | 0.0107 |  | HSP90AA1, HSPA8 |
| Clathrin-mediated Endocytosis Signaling | 1.29 | 0.0104 |  | HSPA8, RAB5B |
| Natural Killer Cell Signaling | 1.27 | 0.0101 |  | HLA-G, HSPA8 |
| HIF1α Signaling | 1.23 | 0.00962 |  | HSP90AA1, HSPA8 |
| Glutathione-mediated Detoxification | 1.17 | 0.027 |  | NAT8B |
| Antigen Presentation Pathway | 1.15 | 0.0256 |  | HLA-G |
| NRF2-mediated Oxidative Stress Response | 1.14 | 0.00844 |  | CCT7, HSP90AA1 |
| Spliceosomal Cycle | 1.05 | 0.0204 |  | HSPA8 |
| Cell Cycle: G2/M DNA Damage Checkpoint Regulation | 1.05 | 0.02 |  | TOP2A |
| Circadian Rhythm Signaling | 1.04 | 0.00746 |  | H3-4, NR1D1 |
| UVB-Induced MAPK Signaling | 1.03 | 0.0192 |  | H3-4 |
| Huntington's Disease Signaling | 1 | 0.00707 |  | HSPA8, PSMD6 |
| Cell Cycle Control of Chromosomal Replication | 1 | 0.0179 |  | TOP2A |
| FAT10 Signaling Pathway | 1 | 0.0179 |  | PSMD6 |
| Primary Immunodeficiency Signaling | 0.979 | 0.0169 |  | DCLRE1C |
| Sirtuin Signaling Pathway | 0.979 | 0.00683 |  | ATG16L1, H3-4 |
| Pyridoxal 5'-phosphate Salvage Pathway | 0.939 | 0.0154 |  | PDXK |
| Synaptogenesis Signaling Pathway | 0.928 | 0.00635 |  | HSPA8, RAB5B |
| Mitotic Roles of Polo-Like Kinase | 0.924 | 0.0149 |  | HSP90AA1 |
| Stearate Biosynthesis I (Animals) | 0.924 | 0.0149 |  | FASN |
| Remodeling of Epithelial Adherens Junctions | 0.921 | 0.0147 |  | RAB5B |
| Hypoxia Signaling in the Cardiovascular System | 0.876 | 0.0132 |  | HSP90AA1 |
| IL-7 Signaling Pathway | 0.863 | 0.0128 |  | EBF1 |
| TR/RXR Activation | 0.836 | 0.0119 |  | FASN |
| Xenobiotic Metabolism AHR Signaling Pathway | 0.821 | 0.0115 |  | HSP90AA1 |
| Unfolded protein response | 0.807 | 0.0111 |  | HSPA8 |
| Crosstalk between Dendritic Cells and Natural Killer Cells | 0.801 | 0.011 |  | HLA-G |
| Virus Entry via Endocytic Pathways | 0.75 | 0.00962 |  | HLA-G |
| NER (Nucleotide Excision Repair, Enhanced Pathway) | 0.75 | 0.00962 |  | TOP2A |
| PD-1, PD-L1 cancer immunotherapy pathway | 0.742 | 0.00943 |  | HLA-G |
| PPAR Signaling | 0.738 | 0.00935 |  | HSP90AA1 |
| Telomerase Signaling | 0.735 | 0.00926 |  | HSP90AA1 |
| Prostate Cancer Signaling | 0.712 | 0.00877 |  | HSP90AA1 |
| Amyotrophic Lateral Sclerosis Signaling | 0.706 | 0.00862 |  | RAB5B |
| Neuregulin Signaling | 0.703 | 0.00855 |  | HSP90AA1 |
| Nitric Oxide Signaling in the Cardiovascular System | 0.693 | 0.00833 |  | HSP90AA1 |
| p38 MAPK Signaling | 0.693 | 0.00833 |  | H3-4 |
| Neuroprotective Role of THOP1 in Alzheimer's Disease | 0.69 | 0.00826 |  | HLA-G |
| LXR/RXR Activation | 0.684 | 0.00813 |  | FASN |
| FXR/RXR Activation | 0.674 | 0.00794 |  | FASN |
| SNARE Signaling Pathway | 0.646 | 0.00735 |  | HSPA8 |
| White Adipose Tissue Browning Pathway | 0.64 | 0.00725 |  | KLB |
| NAD Signaling Pathway | 0.606 | 0.00662 |  | HSPD1 |
| Type I Diabetes Mellitus Signaling | 0.606 | 0.00393 |  | HLA-G, HSPD1 |
| Aryl Hydrocarbon Receptor Signaling | 0.587 | 0.00629 |  | HSP90AA1 |
| Inhibition of ARE-Mediated mRNA Degradation Pathway | 0.578 | 0.00617 |  | PSMD6 |
| HOTAIR Regulatory Pathway | 0.577 | 0.00613 |  | H3-4 |
| Ribonucleotide Reductase Signaling Pathway | 0.561 | 0.00588 |  | H3-4 |
| Tumor Microenvironment Pathway | 0.542 | 0.00559 |  | HLA-G |
| Regulation of eIF4 and p70S6K Signaling | 0.538 | 0.00552 |  | EIF3F |
| IL-17 Signaling | 0.526 | 0.00535 |  | HSP90AA1 |
| Xenobiotic Metabolism CAR Signaling Pathway | 0.519 | 0.00524 |  | HSP90AA1 |
| Xenobiotic Metabolism PXR Signaling Pathway | 0.517 | 0.00521 |  | HSP90AA1 |
| PI3K/AKT Signaling | 0.502 | 0.005 |  | HSP90AA1 |
| mTOR Signaling | 0.478 | 0.00467 |  | EIF3F |
| Systemic Lupus Erythematosus Signaling | 0.478 | 0.00317 |  | HLA-G, LSM7 |
| ERK/MAPK Signaling | 0.476 | 0.00465 |  | H3-4 |
| Autophagy | 0.475 | 0.00463 |  | ATG16L1 |
| Multiple Sclerosis Signaling Pathway | 0.465 | 0.0045 |  | HLA-G |
| EIF2 Signaling | 0.457 | 0.00441 |  | EIF3F |
| AMPK Signaling | 0.435 | 0.00413 |  | FASN |
| Xenobiotic Metabolism Signaling | 0.373 | 0.00344 |  | HSP90AA1 |
| Neuroinflammation Signaling Pathway | 0.345 | 0.00315 |  | HLA-G |
| Estrogen Receptor Signaling | 0.268 | 0.00244 |  | HSP90AA1 |
| Protein Kinase A Signaling | 0.267 | 0.00244 |  | H3-4 |
| Cytotoxic T Lymphocyte-mediated Apoptosis of Target Cells | 0.256 | 0.00235 |  | HLA-G |
| Graft-versus-Host Disease Signaling | 0.243 | 0.00224 |  | HLA-G |
| Autoimmune Thyroid Disease Signaling | 0.236 | 0.00218 |  | HLA-G |
| OX40 Signaling Pathway | 0.223 | 0.00209 |  | HLA-G |
| Th17 Activation Pathway | 0.22 | 0.00207 |  | HSP90AA1 |
| Allograft Rejection Signaling | 0.218 | 0.00205 |  | HLA-G |
| Axonal Guidance Signaling | 0.208 | 0.00196 |  | EIF3F |
| NUR77 Signaling in T Lymphocytes | 0.205 | 0.00195 |  | HLA-G |
| CTLA4 Signaling in Cytotoxic T Lymphocytes | 0 | 0.00164 |  | HLA-G |
| Dendritic Cell Maturation | 0 | 0.00168 |  | HLA-G |
| Communication between Innate and Adaptive Immune Cells | 0 | 0.00107 |  | HLA-G |
| CDC42 Signaling | 0 | 0.00173 |  | HLA-G |
| B Cell Receptor Signaling | 0 | 0.00157 |  | EBF1 |
| T Cell Receptor Signaling | 0 | 0.00162 |  | HLA-G |
| T Cell Exhaustion Signaling Pathway | 0 | 0.00176 |  | HLA-G |
| Systemic Lupus Erythematosus in T Cell Signaling Pathway | 0 | 0.00155 |  | HLA-G |
